# FAST MS: Software for the Automated Analysis of Top-Down Mass Spectra of Polymeric Molecules Including RNA, DNA, and Proteins

**DOI:** 10.1021/jasms.4c00236

**Published:** 2024-12-23

**Authors:** Michael Palasser, Kathrin Breuker

**Affiliations:** Institute of Organic Chemistry and Center for Molecular Biosciences Innsbruck (CMBI), University of Innsbruck, 6020 Innsbruck, Austria

## Abstract

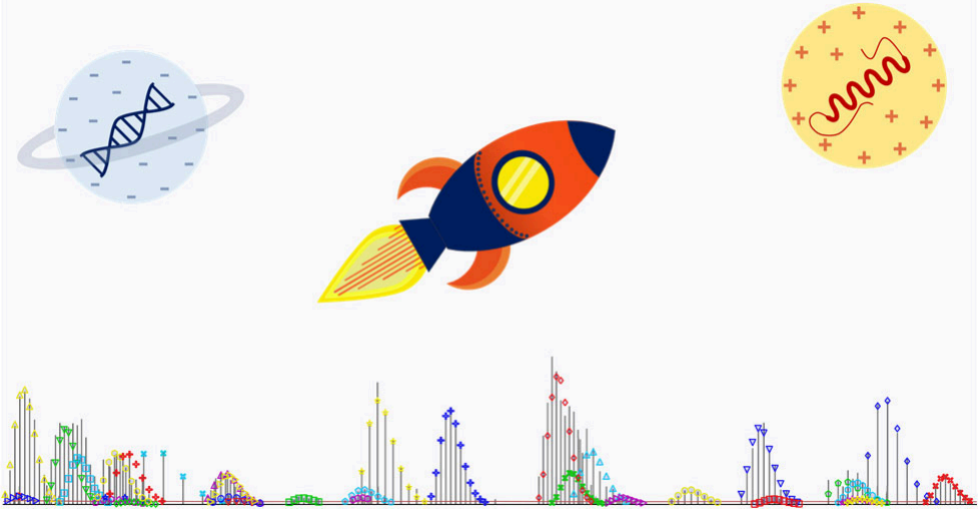

Top-down mass spectrometry (MS) enables comprehensive characterization of modified proteins and nucleic acids and, when native electrospray ionization (ESI) is used, binding site mapping of their complexes with native or therapeutic ligands. However, the high complexity of top-down MS spectra poses a serious challenge to both manual and automated data interpretation, even when the protein, RNA, or DNA sequence and the type of modification or the ligand are known. Here, we introduce FAST MS, a user-friendly software that identifies, assigns and relatively quantifies signals of molecular and fragment ions in MS and MS/MS spectra of biopolymers with known sequence and provides a toolbox for statistical analysis. FAST MS searches mass spectra for ion signals by comparing all signals in the spectrum with isotopic profiles calculated from known sequences, resulting in superior sensitivity and an increased number of assigned fragment ions compared to algorithms that rely on artificial monomer units while maintaining the false positive rate on a moderate level (<5%). FAST MS is an open-source, cross-platform software for the accurate identification, localization and relative quantification of modifications, even in complex mixtures of positional isomers of proteins, oligonucleotides, or any other user-defined linear polymer.

## Introduction

Top-down mass spectrometry (MS) is a powerful approach for the identification, localization, and relative quantification of posttranslational,^1−3^ posttranscriptional,^4^ and synthetic modifications,^5^ and has recently been extended to the analysis of products from reactions for chemical probing of ribonucleic acid (RNA) structure^6^ and binding site mapping of RNA-peptide^7,8^ and RNA-small molecule^9,10^ complexes. By omitting the digestion step characteristic of bottom-up MS and instead dissociating intact molecules in the gas phase, possible correlations between posttranslational or posttranscriptional modifications (PTMs) are preserved in top-down MS, allowing for full characterization of ’proteoforms’^11^ or different isomers and forms of RNA.^12^ Recent advances in instrumentation, along with the increasing number of FDA-approved protein, peptide and oligonucleotide therapeutics,^13−15^ have inspired new applications of top-down MS in academic research and the pharmaceutical industry.^16−20^

While MS/MS spectra can often be acquired within a few minutes, data analysis remains the bottleneck for studies on a larger scale. Depending on the dissociation method and the biomolecule under investigation, up to six (proteins: *a*/*x*, *b*/*y*, *c*/*z*) or eight (nucleic acids: *a*/*w*, *b*/*x*, *c*/*y*, *d*/*z*) different types of fragments can be formed for each backbone cleavage site, often in multiple charge states and accompanied by neutral losses.^16,21^ As a result, top-down mass spectra can be highly complex, especially for larger and heterogeneously modified precursor molecules, making their analysis very challenging. More specifically, a single top-down MS spectrum can contain thousands of isotope peaks representing hundreds of often overlapping isotopic distributions that must be identified, quantified, and assigned to possible fragment species before the data can be used to, for example, localize and quantify modifications or to provide information about ligand binding sites.^7−10,12,22^

Over the past few decades, considerable effort has been invested in automating the analysis of MS and MS/MS spectra, including the detection and quantification of isotopic distributions and the assignment of fragment ions. Perhaps the most influential algorithm, THRASH,^23^ detects and quantifies protein fragment ions by identifying sets of isotopic peaks in the spectrum and comparing them to isotopic distributions obtained by linear interpolation between two abundance distributions from a look-up table of abundance distributions calculated for polymers of the artificial monomer unit ’averagine’ whose elemental composition (C_4.9384_H_7.7583_N_1.3577_O_1.4773_S_0.0417_) represents an average of the canonical amino acid residues.^24^ Algorithms including MS-Deconv,^25^ Xtract (Thermo Fisher Scientific), and SNAP (Bruker Daltonics), have adopted this concept for automated data analysis. Additional programs were developed that can analyze protein fragment lists generated by any of these algorithms in terms of sequence coverage and PTMs^26−30^ or conduct a database search to identify and characterize proteins and proteoforms.^31,32^ Recently, multifunctional software packages were published that incorporate several of these algorithms for applications in the field of proteomics.^33−36^ The use of artificial model amino acids is well suited for untargeted approaches but has some avoidable disadvantages when the sequence of the protein under study is already known.^37−39^ First, a random search for isotopic distributions in the spectrum increases the number of false positives, and second, fragment ions of low abundance are often missed or assigned to an incorrect mass or charge. Third, the deviation of mass and relative isotope abundance values calculated from artificial model amino acids from actual values generally increases with decreasing fragment mass for statistical reasons and can become intolerable if rare elements such as bromine are present. For these reasons, the analysis of top-down mass spectra often requires some level of time-consuming manual postprocessing.

In the emerging field of top-down MS of RNA with applications in modification analysis of biologically relevant or therapeutic RNA and in structural biology, there is a similar need for automated data analysis. For example, RNA therapeutics are typically highly modified, and the characterization of their sequence and modifications, as well as the assessment of their purity, is critical. Among the noncommercial software developed for MS of RNA and deoxyribonucleic acid (DNA),^40−50^ we could not find any that takes into account positional isomers and can relatively quantify site-specific modifications.

The data analysis software FAST MS (Free Analysis Software for Top-Down Mass Spectrometry) was developed to address these challenges. Based on the intact sequence of the (bio)molecule under study, the program identifies, assigns and relatively quantifies isotopic distributions in peak lists derived from MS or MS^n^ spectra and provides several tools for statistical analysis. FAST MS works for RNA, DNA, proteins, and any user-defined linear polymer. Monomer units, modifications, fragment ion types, and even elemental isotope abundances can be defined by the user within the easy-to-use graphical user interface (GUI), providing maximum flexibility for targeted MS applications. FAST MS is open-source and available at GitHub (https://github.com/michael-palasser/FAST-MS/releases).

## Experimental Section

The MS and MS/MS spectra of RNAs, proteins, and DNA 1 (Table 1) were previously recorded on a 7 T Fourier transform ion cyclotron resonance (FT-ICR) instrument (Apex Ultra, Bruker, Austria) equipped with an electrospray ionization (ESI) source, a linear quadrupole for ion isolation, a collision cell for collisionally activated dissociation (CAD) or radical transfer dissociation (RTD), and a hollow dispenser cathode for electron capture dissociation (ECD) or electron detachment dissociation (EDD). ESI spectra were obtained by operation of the linear quadrupole in transmission (radiofrequency-only) mode. For CAD, RTD, ECD, or EDD, ions of interest were isolated in the quadrupole and dissociated in the collision (CAD, RTD) or ICR cell (ECD, EDD); peak picking utilized the FTMS algorithm (Bruker, Austria). The MS/MS spectrum of CpG1018 DNA was recorded on a quadrupole time-of-flight (QTOF) instrument (maXis II, Bruker, Germany) by isolation of (M-9H)^9–^ ions in the quadrupole and dissociation in the collision cell; peak picking used the Sum Peak algorithm (Bruker, Germany).

**Table 1:** RNA, DNA, and Proteins Studied

name	length	mass/kDa	sequence
RNA 1	15 nt	4.8	5′-GAAGG UUCGC CUUCG-3′
RNA 2	15 nt	4.8	5′-GAAGG GCAAC CUUCG-3′
RNA 3	17 nt	5.5	5′-GCGAA CCUGhm^5^C GGGUU CG-3′^a^
RNA 4	22 nt	7.0	5′-CGAAG GUUCG CCUUC GCGUC AG-3′
RNA 5	22 nt	7.0	5′-CGUCA GCGAA GGUUC GCCUU CG-3′
NSR RNA	27 nt	8.5	5′-GGCUG CUUGU CCUUU AAUGG UCCAG UC-3′
RRE-IIB-0 RNA	39 nt	12.6	5′-GGUCU GGGCG CAGCG UCAAU GACGC UGACG GUACA GGCC-3′
RRE-I RNA	47 nt	15.2	5′-GGGUU CUUGG GAGCA GCAGG AUUCG UCCUG GCUGU GGAAA GAUAC CC-3′
DNA 1	20 nt	6.0	5′-GCTAC ATTTA TCACG CGCTT-3′
CpG1018 DNA^b^	22 nt	7.1	5′-TGACT GTGAA CGTTC GAGAT GA-3′
ubiquitin	76 aa	8.6	MQIFV KTLTG KTITL EVEPS DTIEN VKAKI QDKEG IPPDQ QRLIF AGKQL EDGRT LSDYN IQKES TLHLV LRLRGG
calmodulin	148 aa	16.7	ADQLT EEQIA EFKEA FSLFD KDGDG TITTK ELGTV MRSLG QNPTE AELQD MINEV DADGN GTIDF PEFLT MMARK MKDTD SEEEI REAFR VFDKD GNGYI SAAEL RHVMT NLGEK LTDEE VDEMI READI DGDGQ VNYEE FVQMM TAK

a hm^5^C = 5-hydroxymethylcytidine.

b DNA with phosphorothioate backbone.

To estimate the number of false positives, the MS/MS spectra of modified and unmodified precursor ions were analyzed using the correct protein, RNA, or DNA sequence and once more with the same settings using an incorrect fake sequence of equal length. The fake sequence for ubiquitin was truncated calmodulin, and the fake sequence for calmodulin was extended ubiquitin. Since there are only four canonical nucleotides, random RNA or DNA sequences would produce many fragments that are isomeric to the correct ones. For example, using a random DNA sequence for analysis of the CAD spectrum of CpG1018 DNA resulted in an unrealistic false positive rate of 33%. Therefore, all-adenosine sequences were used as fake sequences for all RNAs and DNA 1, and a phosphorothiolated 22 nt fake sequence incorporating 20 adenosines and two terminal uridines was used for CpG1018 DNA. The number of identified *c* and *y* ions (CAD of RNA), *b* and *y* ions (CAD of proteins), *c* and *z*^•^ ions (ECD of proteins), *a*, *a*-base, *b*, *c*, *d*, *w*, *x*, *y,z* ions (CAD of DNA), and *d* and *w* ions (EDD of DNA) in the analyses with the correct and the fake sequences were used to calculate the false discovery rate.

### Description of the Algorithm

**Scheme 1:**
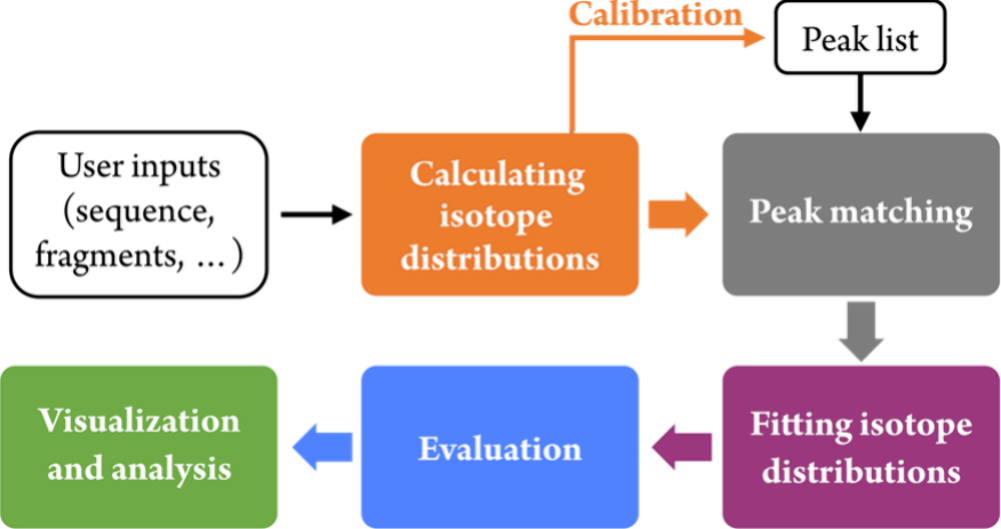
FAST MS Workflow for Analyzing MS/MS Spectra

FAST MS was written in Python 3 using PyCharm 2020 1.4 (JetBrains) and was implemented in a three-tier architecture. It utilizes several open-source Python libraries: Numpy,^51^ Numba,^52^ Pandas,^53^ Scipy,^54^ and XlsxWriter (https://xlsxwriter.readthedocs.io). For creating the GUI, PyQt5 (https://pypi.org/project/PyQt5/), PyQtGraph (https://www.pyqtgraph.org/), and Matplotlib (https://matplotlib.org/) were employed. Furthermore, PyInstaller (https://pyinstaller.org/en/stable/) was used to generate executables that need no Python installation. The main functions and methods were unit-tested using pytest (https://docs.pytest.org/en/stable/contents.html) with total line coverage of >60%. The basic workflow is illustrated in Scheme 1. FAST MS imports experimental data as a peak list in plain text or CSV file format. This list of *m*/*z* and associated signal height values can be generated using the MS instrument manufacturer’s software, e.g. DataAnalysis (Bruker Daltonics) or Xcalibur (Thermo Scientific). No further data reduction (e.g., deconvolution or charge assignment) is required, as FAST MS does not use deconvoluted data but directly compares calculated isotopic distributions of ions with the peak list of an *m*/*z* spectrum.

### Calculation of Theoretical Isotope Distributions

Instead of using artificial monomer units,^23,24,39^ FAST MS calculates exact theoretical isotope distributions based on the chemical formula of each species. Isotopic fine structures are computed by applying the polynomial approach.^55^ Isotope peak masses and abundances are then calculated as the centroid mass and the sum of the isobaric isotopologue abundances. Because this process can be computationally highly demanding for heavier isotope peaks, FAST MS uses a fast Fourier transform (FFT) based method.^55^ The differences between isotope mass values are approximated by
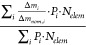
1where *P*_i_ is the abundance of the isotope *i*, *N*_elem_ is the number of atoms of the corresponding element incorporated in the molecule, and Δ*m*_i_ and Δ*m*_nom,i_ describe the exact and nominal mass increases of the isotope relative to its monoisotopic mass; this approach is similar to that proposed by Sadygov.^56^ The mass differences between approximated and centroid mass were typically <0.05 ppm for RNA and <0.20 ppm for proteins.

### Ion Detection

To avoid false positives, FAST MS only searches for the most probable net charges of each fragment, which depend on the precursor ion net charge, the size of the fragment, and any charged modifications. Starting with the most abundant peaks of each theoretical isotope distribution, the program matches the theoretical data with the peak list from the spectrum. Considering that the mass accuracy often decreases with *m*/*z*, a linear mass error threshold is applied:
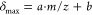
2where *a* and *b* are user-definable constants. Because FAST MS processes peak data instead of the profile spectrum, conventional noise detection workflows are not applicable. Therefore, a new algorithm had to be implemented in which the noise in the *m*/*z* window around an ion signal is determined iteratively by computing the mean intensity within the user-definable window (default width: 3 *m*/*z*), excluding peaks that are more than 33% higher than the mean value, and calculating the mean again. This procedure is repeated until the mean remains constant. Depending on the remaining peak density ρ in the window, this mean value is multiplied by the factor
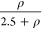
3(for ρ > 5) or by a factor of 0.67 (otherwise) to obtain noise values for each window, which increases the accuracy in regions of the spectrum where the peak density is high enough for this correction. The proposed workflow was developed empirically and worked for different analytes, ion polarities, dissociation techniques, and types of mass spectrometers (Figure S1). Because it relies on the presence of noise peaks in the list of peaks fed into FAST MS, the thresholds used for the peak picking algorithm (e.g., FTMS in DataAnalysis) should be sufficiently low. Otherwise, FAST MS might consider actual peaks as noise, leading to inaccurate S/N values or even missed ion signals.

### Ion Quantification and Evaluation

The basic steps in the quantification process are illustrated in Figure S2. The theoretical abundances of the isotope peaks are iteratively fitted to the observed abundances using a least-squares model. Individual isotope peaks that deviate from a given theoretical isotopic distribution (’outliers’) are identified using an empirically adapted version of the Grubbs test.^57^ When signals of different ions overlap in *m*/*z*, a linear combination of the corresponding isotopic distributions is modeled in the next step. To avoid overfitting and false positives, overlapping ions are deleted when their abundance decreases to a user-definable percentage of the previous abundance during an iteration step (default: 80/number of distributions in %, e.g., 40% for two overlapping distributions). After the fit, the program calculates a quality error value for each ion, which is defined by the minimum of the fit’s cost function divided by the intensity of the ion or those of overlapping ions. The signal-to-noise ratio (S/N) is calculated by dividing the intensity (sum of the modeled isotope peak signal heights) by the noise next to the ion. The mass and quality errors as well as the S/N values are evaluated, and entries above (errors) or below (S/N) user-defined thresholds are moved to the list of deleted ions.

### Statistical Analysis

Relative ion abundances are calculated by adding the signal heights of all isotopic peaks (reported as “intensity”). For charge-sensitive analyzers (e.g., FT-ICR and Orbitrap), FAST MS includes the option to divide these values by the charge of the ions (reported as “int./z”). Since dissociation of a molecular ion typically produces two complementary fragment ions, fragment (but not molecular) ion abundances are halved for calculation of relative fragment yields. Sequence coverage is calculated as the percentage of the number of cleavage sites for which fragments were observed relative to the total number of cleavage sites.

## Results and Discussion

FAST MS was designed to provide flexibility for analyzing any type of linear polymers and modifications. In addition to amino acids and nucleotides, new monomer units and post-translational, post-transcriptional, or synthetic modifications can be defined by the user. Importantly, FAST MS allows for the analysis of mixtures of positional isomers, including therapeutic peptides, proteins, and RNA with heterogeneous modification profiles (an example is shown in Figure S3). Moreover, isotopically labeled, enriched, or depleted molecules can be analyzed by editing the isotope data. The fragment types that are generated by MS/MS not only depend on the dissociation technique used (e.g., CAD, ECD/ETD, or UVPD)^16^ and the type of molecule but also on the applied energy^58−60^ and modifications (e.g., 2′-modification of RNA).^60,61^ To accommodate all types of MS/MS applications, FAST MS can be customized according to the dissociation technique used and the compound class. For maximum convenience and runtime stability, sequences, modifications, fragment types, monomer units, and isotope data can be easily accessed and edited within the GUI.

### Analyzing an MS/MS Spectrum

**Figure 1:**
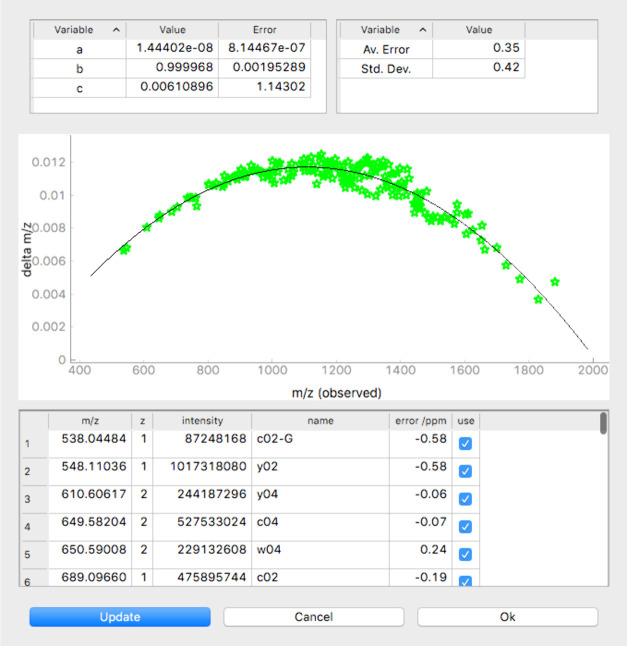
FAST MS dialogue window for internal calibration. FAST MS compares the monoisotopic *m*/*z* values of the signals specified in the list of signals to be used for calibration with the *m*/*z* values of fragment ions calculated from the sequence and calculates fit coefficients, the average mass error, and the standard deviation of the mass errors (both in ppm). The difference between calibrated and uncalibrated *m*/*z* values (Δ*m*/*z*) of the signals and the fit function are visualized graphically, and the user can interactively control the quality of the fit by excluding misassigned signals. Once the user is satisfied with the fit, the program applies the calibration to the peak list for further analysis.

To demonstrate the features of FAST MS, we used data from chemical probing of the 27 nt NSR RNA (Table 1) with CMCT (*N*-Cyclohexyl-*N*′-β-(4-methylmorpholinium)ethylcarbodiimide *p*-toluenesulfonate).^6^ Briefly, the CMC^+^ cation (*N*-Cyclohexyl-*N*′-β-(4-methylmorpholinium)ethylcarbodiimide) reacts with N3 and N1 of exposed uridine and guanosine bases, respectively. To localize the sites and the site-specific extent of CMC^+^-modification, singly CMC^+^-modified NSR ions with a net charge of 7-, (NSR^CMC+^-8H)^7–^, were isolated in the mass spectrometer and dissociated by CAD. Analysis of the CAD data using FAST MS then allows conclusions to be drawn about the structure of the NSR RNA in solution.^6^ At the beginning of the analysis, the user defines the sequence of the molecule, the precursor net charge, the dissociation technique, the type and number of modifications, and the file path of the peak list. In an optional step, the mass spectrum can be calibrated internally by using a tab-separated list that specifies the measured monoisotopic *m*/*z*, z, and intensity values of the signals to be used for calibration. This list can be generated using a data reduction algorithm, such as SNAP, or created manually by selecting monoisotopic signals from the peak list of the spectrum. The algorithm involves several cycles of calibration and rejection of values with unrealistic mass errors until the standard deviation between theoretical and calibrated *m*/*z* values falls below a user-defined threshold. Although this process can run entirely automated, it is possible to manually select or remove ions from the list and recalculate the quadratic calibration function (Figure 1):
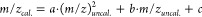
4where *a*, *b*, and *c* are the fit coefficients and *m*/*z*_cal._ and *m*/*z*_uncal._ are the calibrated and uncalibrated *m*/*z* values.

**Figure 2:**
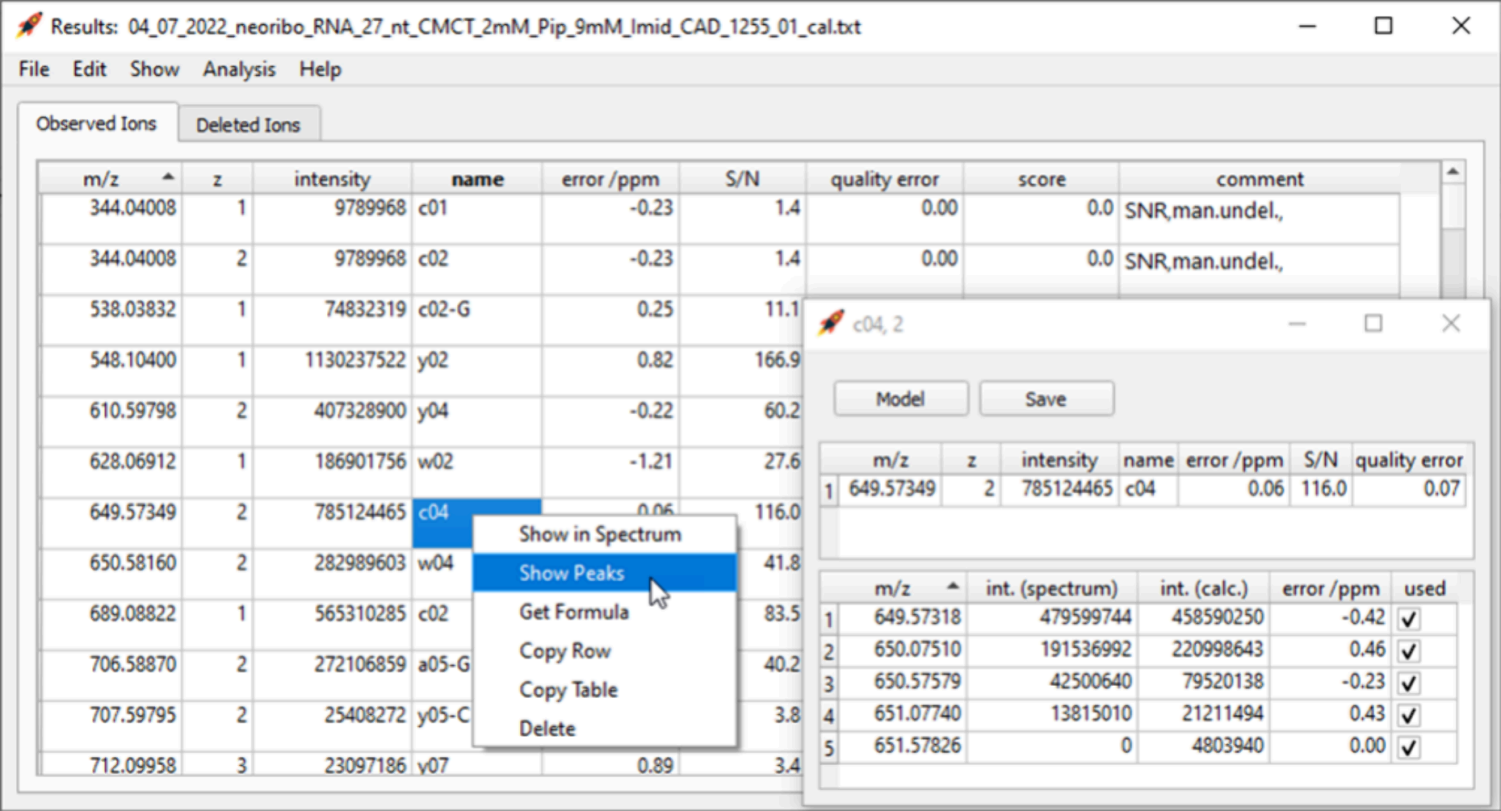
FAST MS window displaying the list of ions identified in a spectrum. Several options are available by right-clicking on an entry. The window on the right shows values related to the isotope peaks of the *c*_4_^2–^ ion. After adjusting the measured peak signal heights (“int. (spectrum)”), the isotope peaks can be refitted (button “Model”).

**Figure 3:**
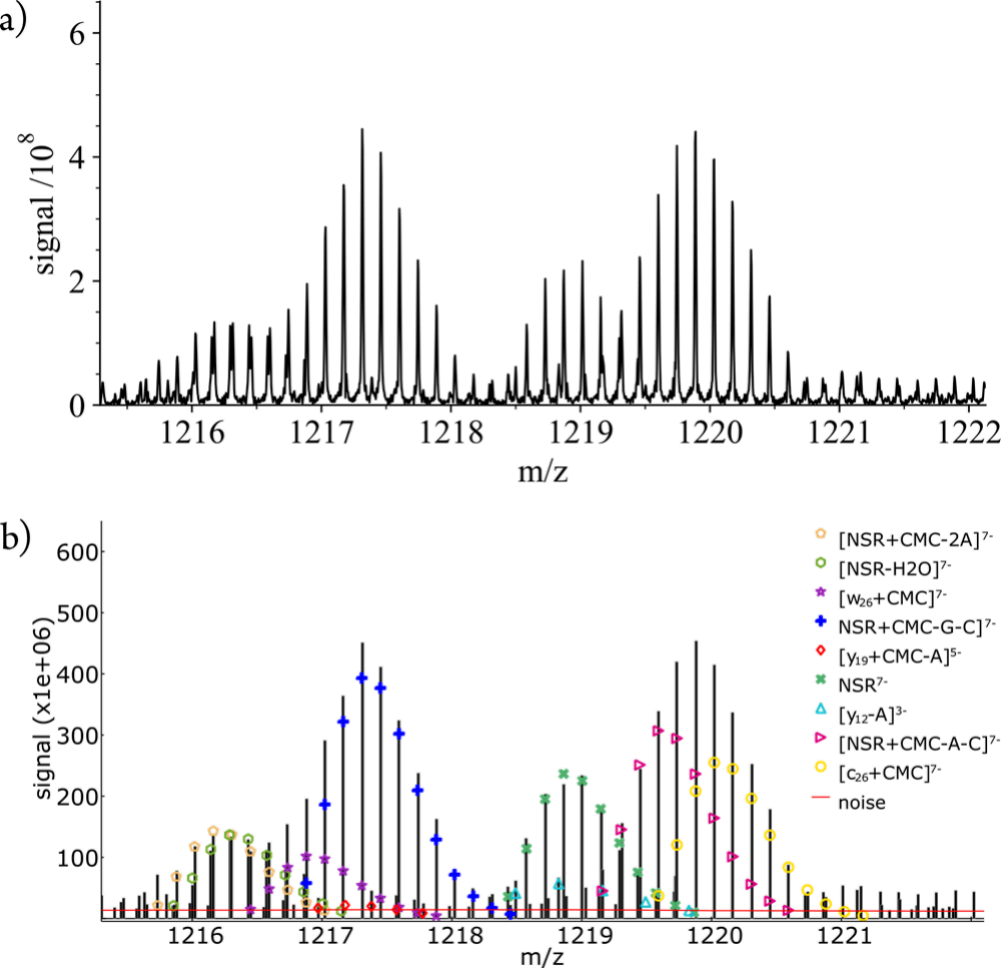
(a) Section of a profile spectrum from CAD of (NSR^CMC+^-8H)^7–^ ions and (b) 9 different overlapping isotopic profiles (markers) from fitting the corresponding peak values (vertical black lines) with FAST MS; the calculated noise level is shown as a horizontal red line.

The assigned and deleted ion signals found by FAST MS are displayed in a list (Figure 2). Overlaps with other ions, the reason for the deletion, and editing events by the user are indicated in the comment section for each ion. The program also highlights suspicious values that might suggest an incorrect assignment. To assist the user while inspecting the results, the isotope peaks can be listed and displayed in an interactive line spectrum. Figure 3 shows that even heavily overlapping isotope clusters are automatically resolved by FAST MS. However, isotope peaks can be manually refitted, deleted, and restored if the user decides that the results need to be corrected. To ensure reproducibility, an audit trail window records the configurations, all inputs and editing events by the user. For later use, the analysis can be saved to a database or exported to Excel.

**Figure 4:**
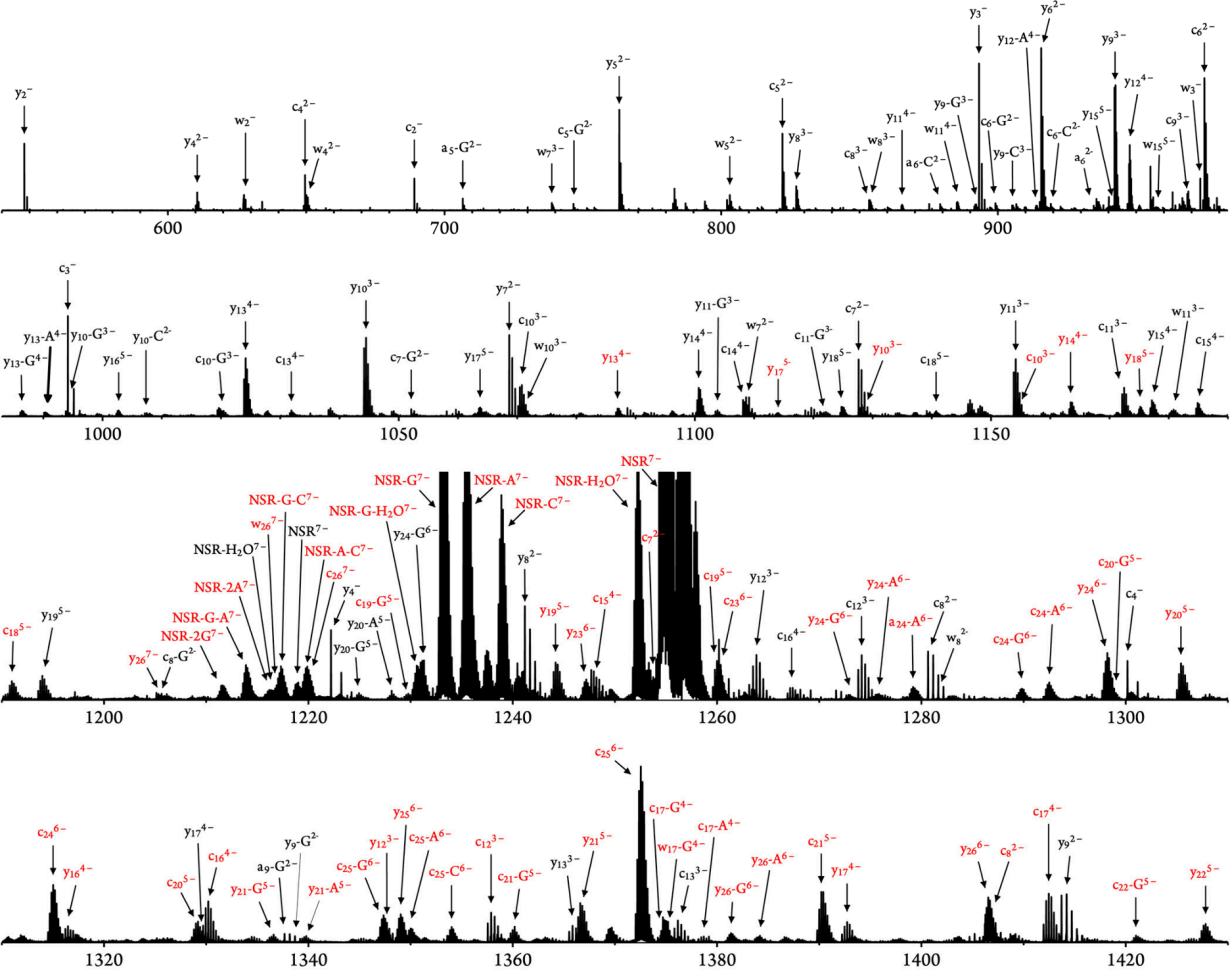
Spectrum from CAD of (NSR^CMC+^-8H)^7–^ ions with red labels for CMC^+^-modified fragments and black labels for unmodified fragments; for clarity, only a fraction (∼30%) of the ions identified by FAST MS (Table S1) are labeled.

Figure 4 shows sections of the assigned CAD spectrum of the (NSR^CMC+^-8H)^7–^ ions. Even though the size of the RNA is only 27 nt, the peak density is high, especially in the *m*/*z* region around the precursor ions. In the *m*/*z* range of 1200–1300 alone, 79 different isotopic distributions were identified. Depending on the secondary structure of the RNA and its sequence, chemical probing typically leads to mixtures of positional isomers from modification at different exposed sites. As a consequence, fragment ions from a given cleavage site can often be observed both with (red) and without (black) CMC^+^-modification (e.g., *y*_10_). In addition, many fragments occur in more than one charge state, and some nucleobase loss caused by CAD further increases the complexity of the spectrum. It is easy to imagine that manual assignment and quantification would take hours or even days of tedious work. Using FAST MS, however, this work can usually be done within a few minutes.

### Evaluation

To evaluate the performance of FAST MS, the spectrum shown in Figure 4 was analyzed by both FAST MS and SNAP. Similar to THRASH, SNAP uses artificial monomer units to detect ion signals in the spectrum. Using a relative intensity threshold of 0.01%, SNAP found 616 ions, of which 261 could be assigned. When the relative intensity threshold was lowered to 0.001%, 1504 ions were detected, but still only 286 could be assigned. Apparently, lowering the threshold leads to an increased number of false positives without significantly increasing the number of assigned ions. Using a comparably low S/N threshold of 2, FAST MS assigned 482 ions (437 correctly from manual inspection; Table S1) in the same spectrum, which highlights the benefit of searching for isotope distributions calculated from the known sequence.

**Figure 5:**
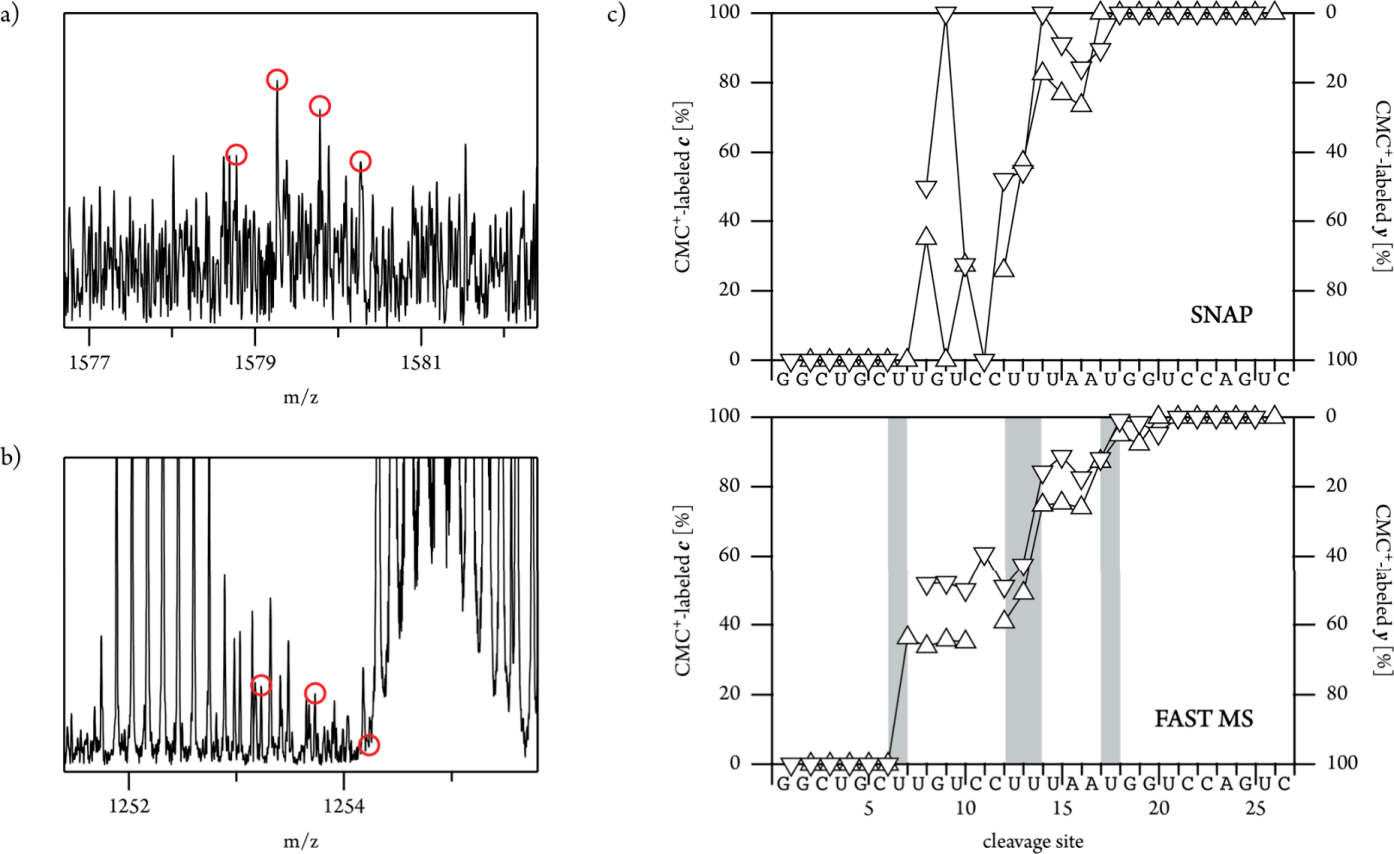
Signals of (a) (*c*_9_^CMC+^-3H)^2–^ and (b) (*c*_7_^CMC+^-3H)^2–^ in a spectrum from CAD of (NSR^CMC+^-8H)^7–^ ions (with lower S/N compared to the spectrum in Figure 4 and for which FAST MS identified 173 ions; see Table S2) which were missed by the SNAP algorithm; red circles indicate the isotope peaks calculated by FAST MS. (c) Site-specific fractions of CMC^+^-modified *c* (upward triangles, left axis) and *y* (downward triangles, right axis) fragments versus cleavage site from analysis of the same spectrum using SNAP (top) and FAST MS (bottom). Site-specific fractions of CMC^+^-modified *c* or *y* fragments were calculated relative to all *c* or *y* fragments from a given cleavage site.^6,7,10^

To further test FAST MS, five CAD spectra of modified NSR and RRE-IIB RNA^7^ were analyzed by both SNAP and FAST MS (Table S2). On average, FAST MS found more than twice as many *c* and *y* ions as SNAP. We attribute this to the fact that algorithms based on artificial monomer units do not search for specific fragment ions, which makes them more likely to miss ions of lower abundance (Figure 5a), especially in crowded regions of the spectrum (Figure 5b). However, for the analysis of modification profiles or ligand binding, low abundance ions can be very important. More generally, the detection of ions with low S/N is critical when molecules are large,^37^ present at low concentrations, and/or when recording time is limited (LC-MS/MS). Figure 5c, in which the site-specific fractions of CMC^+^-modified *c* and *y* fragments are plotted versus cleavage site, illustrates the result of missed isotopic profiles. RNA backbone cleavage produces pairs of complementary fragments, so the fractions of CMC^+^-modified *c* and *y* fragments should add up to 100% for each cleavage site, and they should increase with increasing size of the fragments.^6^ Because ions with low S/N (including CMC^+^-modified *c*_7_^2–^ and *c*_9_^2–^) were missed by SNAP, the corresponding data show relatively large scatter, making it difficult to accurately assign the modification sites. In contrast, the data obtained by use of FAST MS clearly reveal CMC^+^-modification of U7, U13, U14 and U18.

**Figure 6:**
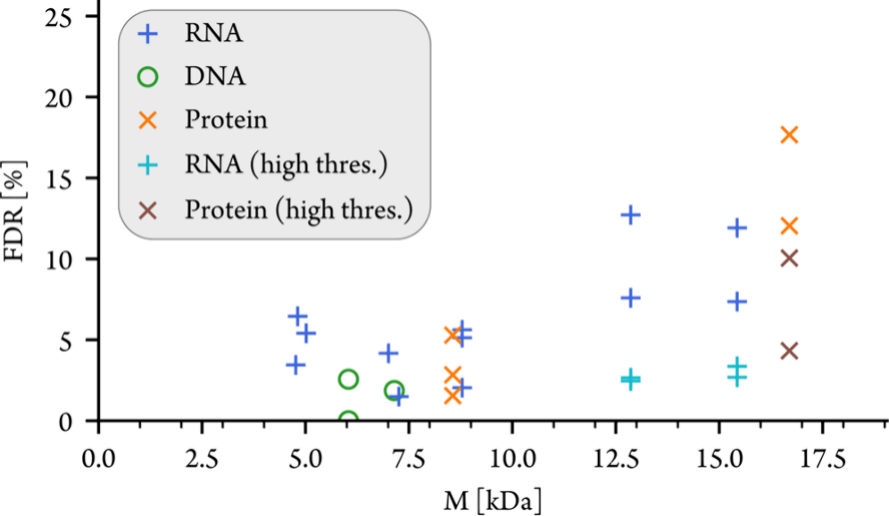
False discovery rate (FDR) versus precursor ion mass M from analyses of MS/MS spectra by FAST MS. Thresholds of 0.5 (quality error) and 2 or 3 (S/N) were used for FT-ICR spectra of RNA/DNA or proteins, respectively. Due to the lower resolving power of the QTOF, thresholds of 5 (S/N) and 0.3 (quality error) were used for CpG1018. MS/MS spectra of precursor ion masses above 10 kDa were processed a second time with the higher thresholds (“high thres.”) of 5 (S/N) and 0.3 (quality error).

Manual inspection of the assigned ions in these five CAD spectra (Table S2) identified on average 5% as false positives. In the course of a more standardized approach, 20 MS/MS spectra were analyzed in a statistical study similar to a target-decoy approach (Table S3). Although FAST MS was not developed for protein identification and database searches, an approach similar to traditional target-decoy studies in bottom-up proteomics^62^ can be used to estimate the specificity of the algorithm. For this purpose, each spectrum was analyzed twice by FAST MS, once with the correct sequence that produces both true (TP) and false (FP) positive assignments and once with an incorrect fake sequence that should ideally produce only false positive (FP) assignments. The ratio FP/(TP+FP) then provides a good approximation of the false discovery rate (FDR).^62^ The evaluated spectra included 14 CAD spectra of RNA and DNA, an EDD spectrum of DNA, and one CAD spectrum and four ECD spectra of proteins (Table S3). Figure 6 shows that the results of FAST MS are highly accurate for oligonucleotides and proteins with up to ∼10 kDa (3.4% FDR on average). The increased FDR for larger molecules is a general phenomenon, not specific to FAST MS, and can be attributed to the increased complexity of their MS/MS spectra as a result of broader isotope distributions and higher numbers of cleavage sites that lead to more isotope peaks and fragment ions, respectively. Increasing the S/N and quality error thresholds for the larger precursor ions significantly reduced the false discovery rate, while moderately decreasing the number of ions found by ∼17%. However, keeping both the FDR low and the number of ions found high for larger molecules would require improved spectral quality and thus spectrometers with higher mass resolving power and sensitivity.

### Analyzing the Ion List

**Figure 7:**
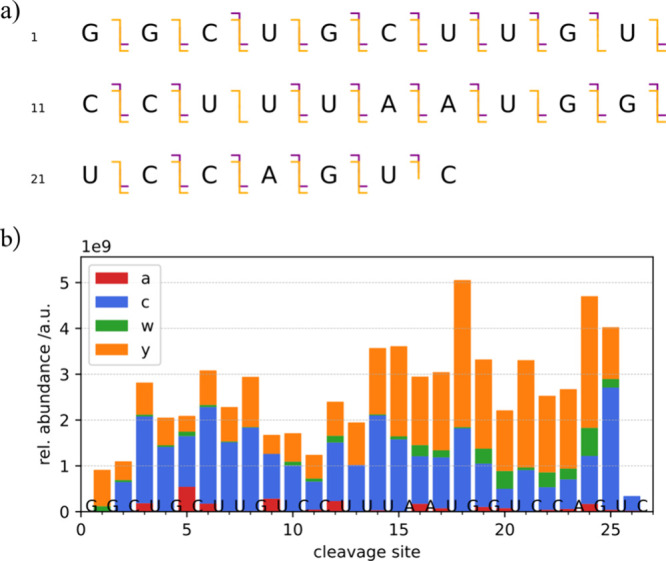
FAST MS windows illustrating the statistical analysis of fragment ions from CAD of (NSR^CMC+^-8H)^7–^ ions: (a) fragment ion map illustrating sequence coverage (*a* fragments purple, *c* orange, *w* purple, *y* orange); (b) relative fragment ion abundances versus cleavage site; total fragment yields were 1% (*a*), 10% (*c*), 1% (*w*) and 10% (*y*).

Processing a spectrum to generate an ion list is typically just the starting point of data evaluation. For further analysis, FAST MS provides several tools to assist the user in generating meaningful results. For example, the sequence coverage can be determined and graphically displayed in an interactive fragment cleavage map (Figures 7a, S4). Monitoring fragment yields is not only relevant when evaluating instrument or experiment parameters.^59,63^ It has also proven critical to better understand the effect of nucleobases,^60,64^ net charge,^60,65^ modifications,^12,60,66^ and noncovalent interactions^67−69^ on RNA or protein dissociation. For these and other purposes, FAST MS can automatically calculate site-specific relative fragment ion abundances (Figure 7b). To investigate the effect of negative or positive charges of modifications, the average charge can also be determined for each fragment.

**Figure 8:**
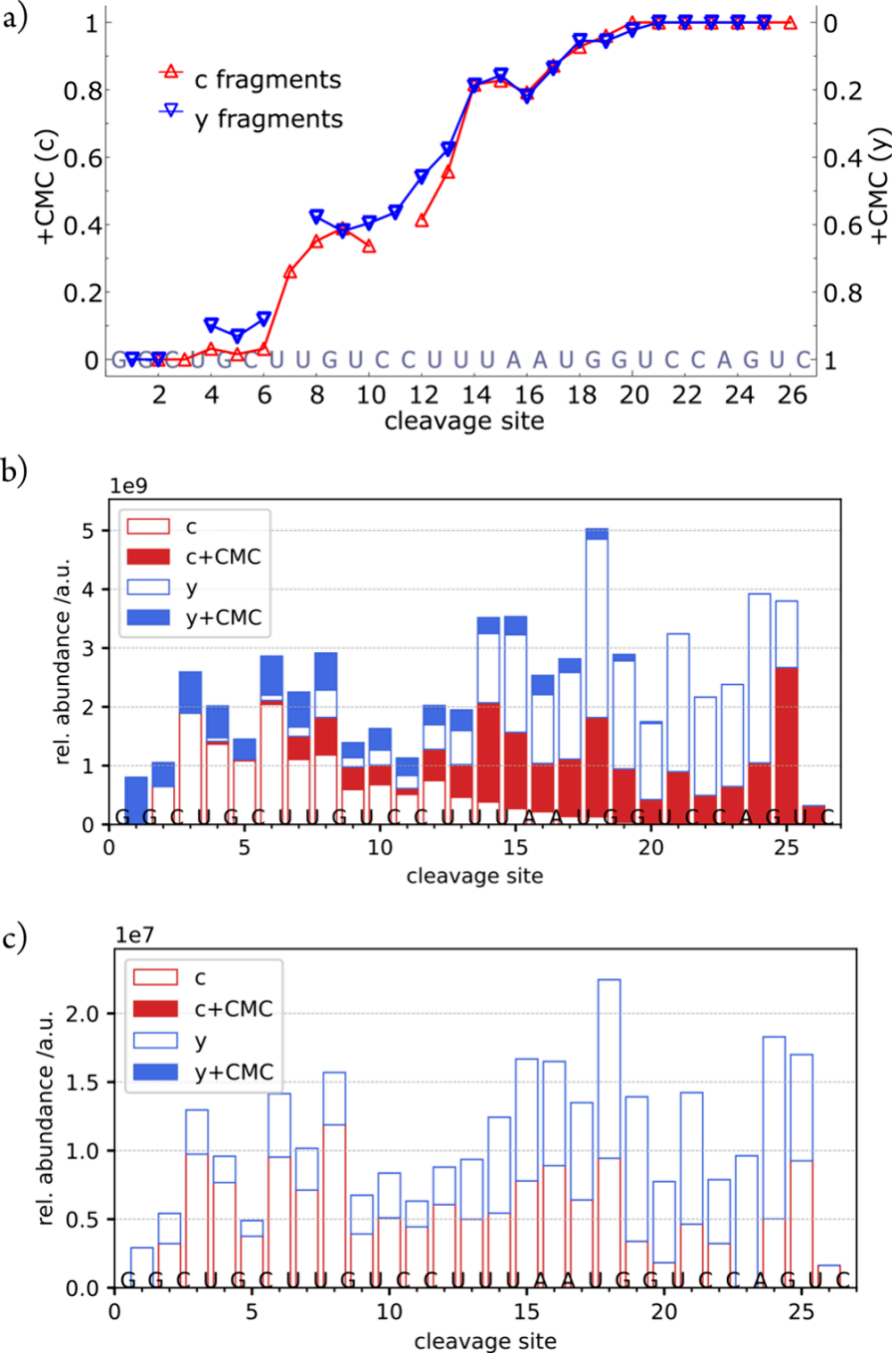
FAST MS plots displaying (a) CMC^+^-modified fractions and (b) relative abundances of *c* (red) and *y* (blue) fragments from CAD of singly modified (NSR^CMC+^-8H)^7–^ ions; (c) relative abundances of *c* and *y* fragments of (NSR-7H)^7–^ ions versus cleavage site.

The most important applications of top-down MS involve the localization of modifications of molecules or ligand binding sites in noncovalent complexes. There are already several programs that can localize modifications qualitatively. However, to characterize mixtures of positional isomers,^12^ heterogeneous products from chemical probing reactions^6,70−72^ or multiple ligand binding sites,^7,9,10^ accurate relative quantification of fragments with and without modification or ligand attachment is required. To this end, FAST MS provides a tool that displays the data graphically and can be used to relatively quantify the site-specific extent of modification or ligand attachment. As in Figure 5, the plot in Figure 8a allows for the relative nucleotide reactivities in a chemical probing experiment to be determined. Moreover, the relative abundances of modified and unmodified fragments (Figure 8b) provide a visualized measure of the significance of the data points in Figure 8a. We used this feature to test the idea that the fixed positive charge of the CMC^+^ modification alters the fragmentation behavior of the RNA but comparing the data from CAD of unmodified (NSR-7H)^7–^ ions (Figure 8c) with those of the (NSR^CMC+^-8H)^7–^ ions (Figure 8b) showed no significant differences.

The above examples show how FAST MS can be used for the characterization of products from RNA chemical probing reactions, and the same approach can be used for various other applications including the identification of ligand binding sites, the localization of hydrogen–deuterium exchange sites and PTMs (e.g., protein deamidations or oxidations), as well as the analysis of synthetic peptide and oligonucleotide impurities. The ability to localize and relatively quantify modified sites can eliminate the need for liquid chromatography (LC) separations, which are challenging for positional isomers of oligonucleotides, and thus the use of expensive and environmentally problematic LC eluents such as trifluoroacetic acid or hexafluoroisopropanol.^17^

## Conclusions

FAST MS is a user-friendly, cross-platform, and open-source software for the analysis of ESI MS and MS/MS spectra. The approach of searching for defined fragment ions from dissociation of ions of known primary structure instead of using artificial monomer units provides superior sensitivity and accurate relative quantification. FAST MS includes several tools for further analysis of ion lists to extract the desired information from top-down MS experiments. Beyond the analysis of MS/MS spectra, FAST MS incorporates several other functionalities. ESI MS spectra can be processed and analyzed in terms of modifications or ligand binding in thermodynamic studies and for monitoring reaction and ligand binding kinetics. In contrast to most other programs, there are no restrictions concerning molecule type, dissociation methods, ion polarity, monomer units, modifications, ligands, adducts, or isotope labels. Moreover, software parameters and thresholds can be edited within the GUI, which provides the user with maximum flexibility and control. We hope that these features will make data analysis easier for MS users and stimulate new applications of top-down MS.

## Supporting Information Available

Illustration of noise calculation for both FT-ICR and QTOF data, schematic illustration of FAST MS workflow for identifying and quantifying ion signals in a mass spectrum, illustration of the analysis of multiple modifications by FAST MS, fragment ion maps illustrating sequence coverage, list of ions assigned by FAST MS in a spectrum from CAD of (NSR^CMC+^-8H)^7–^ ions, comparison of the performance of FAST MS and SNAP in ion detection, summary of experiments for estimating the FDR of ion assignments by FAST MS (PDF)
