# Supplementary material for: FAST MS: Software for the Automated Analysis of Top-Down Mass Spectra of Polymeric Molecules Including RNA, DNA, and Proteins
Source: J Am Soc Mass Spectrom. 2024 Dec 23;36(2):247–57. doi: 10.1021/jasms.4c00236 (PMC11808778; doi:10.1021/jasms.4c00236)
Supplement: Supplementary file 1 — js4c00236_si_001.pdf [file js4c00236_si_001.pdf]

Supporting Information for

FAST MS: A software for the automated analysis of top-down mass spectra of polymeric molecules including RNA, DNA and proteins

Michael Palasser*† and Kathrin Breuker*

Institute of Organic Chemistry and Center for Molecular Biosciences Innsbruck (CMBI), University of Innsbruck, 6020 Innsbruck, Austria

Corresponding Authors

*Kathrin Breuker, email: kathrin.breuker@uibk.ac.at
*Michael Palasser, email: michael.palasser@bachem.com

Present Address

†Bachem AG, Hauptstrasse 144, 4416 Bubendorf

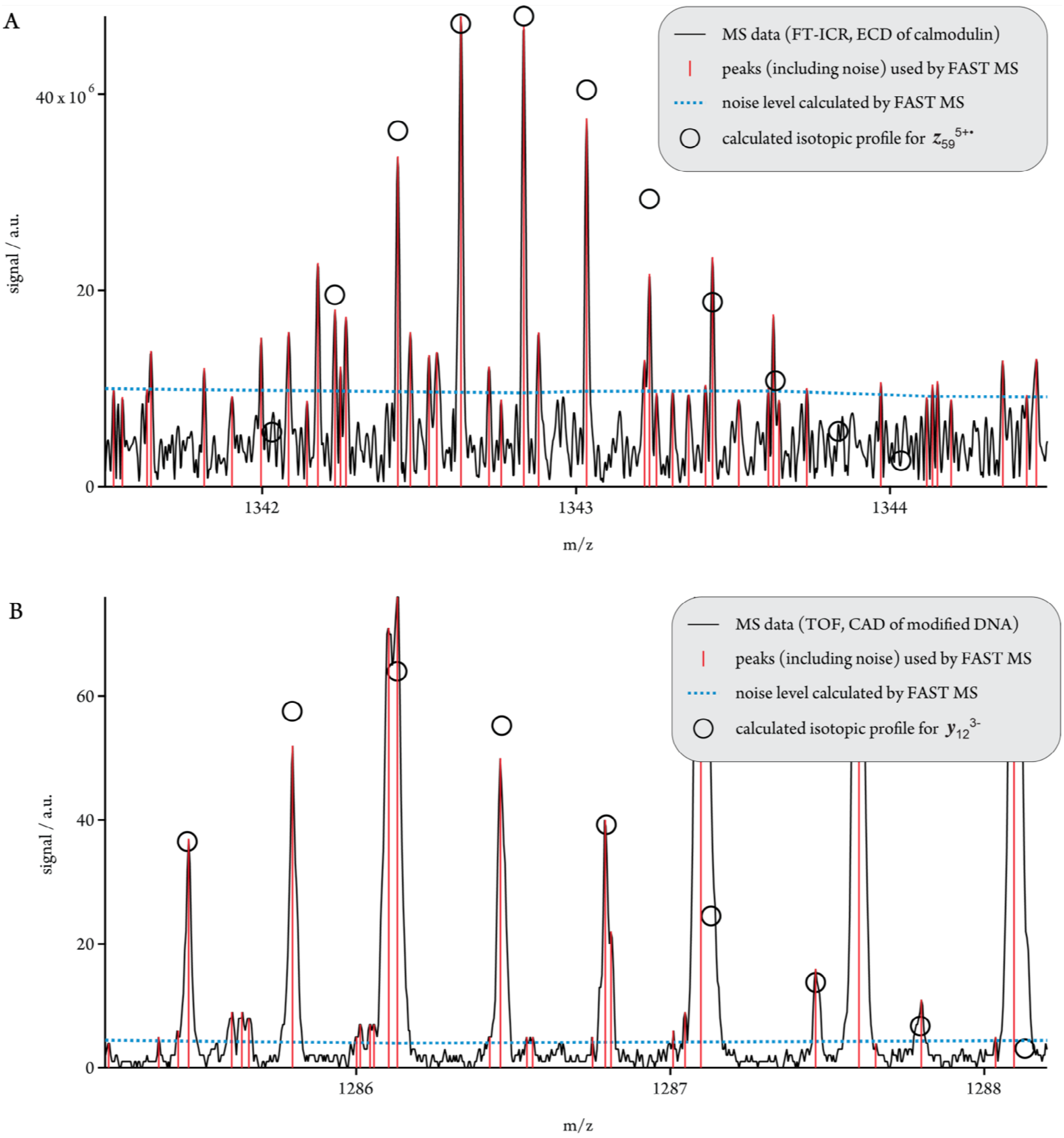

Figure S1: Ion detection by FAST MS works for different analytes, ion polarity, dissociation techniques, and types of mass spectrometers. Peak lists (red lines) that include noise peaks are generated from MS data (black lines) and fed into FAST MS, which uses a least squares model for iteratively fitting theoretical isotope distributions (calculated from the chemical formulae of fragment ions) to the observed abundances (circles) and performs a noise calculation (blue line), illustrated for A) $z_{59}^{5+\bullet}$ fragment ion of calmodulin from ECD using the 7 T FT-ICR instrument and B) y_{12}^{3-} fragment ion of CpG1018 DNA from CAD using the QTOF instrument.

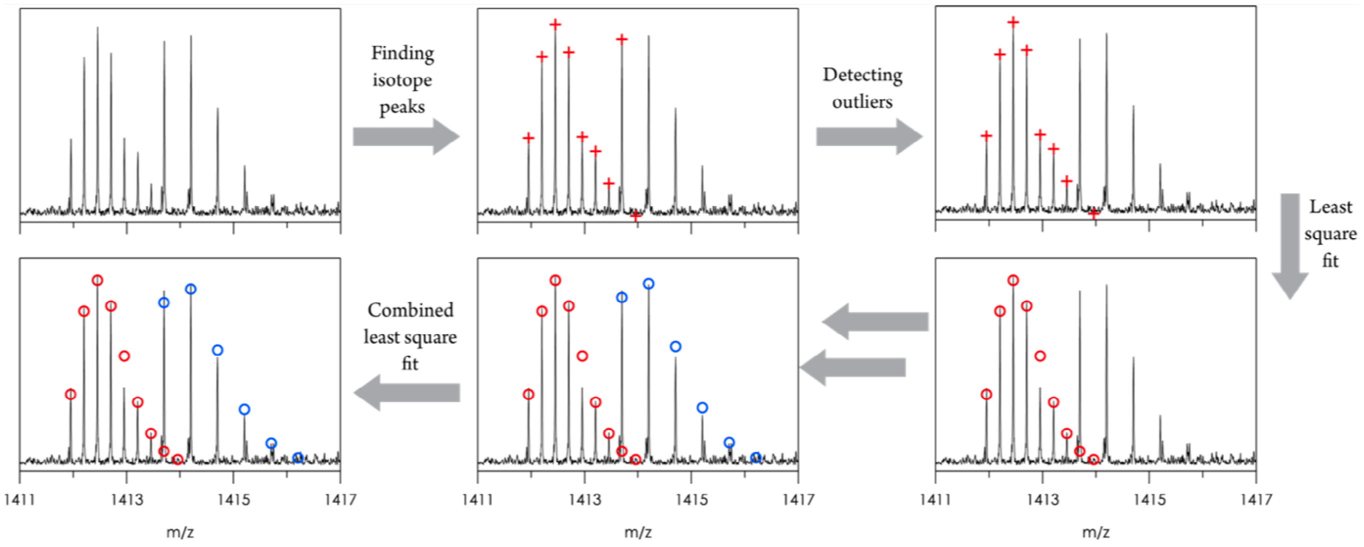

Figure S2: FAST MS workflow for identifying and quantifying ion signals in a mass spectrum, demonstrated for the partly overlapping isotopic distributions of (c_{17}^{CMC+}-5H)$^{4-}$ (red) and y_9^{2-} (blue) of NSR RNA. For all fragment ions predicted from the sequence (including possible modifications), the algorithm calculates theoretical isotopic distributions and searches the peak list for matches in m/z ('Finding isotope peaks'). The theoretical isotopic distributions are fitted to the observed signals (red crosses) and outliers are detected using an empirically adapted version of the Grubbs test ('Detecting outliers'). Outliers are disregarded in the initial least squares fitting procedure ('Least square fit'). Following the initial assignment of ion signals, the program searches for peaks matched to more than one ion (e.g. the outlier of the (c_{17}^{CMC+}-5H)$^{4-}$ ions and the monoisotopic peak of the y_9^{2-} ions). All overlapping isotope peak clusters are re-fitted using a linear combination of the corresponding theoretical isotopic distributions ('Combined least square fit').

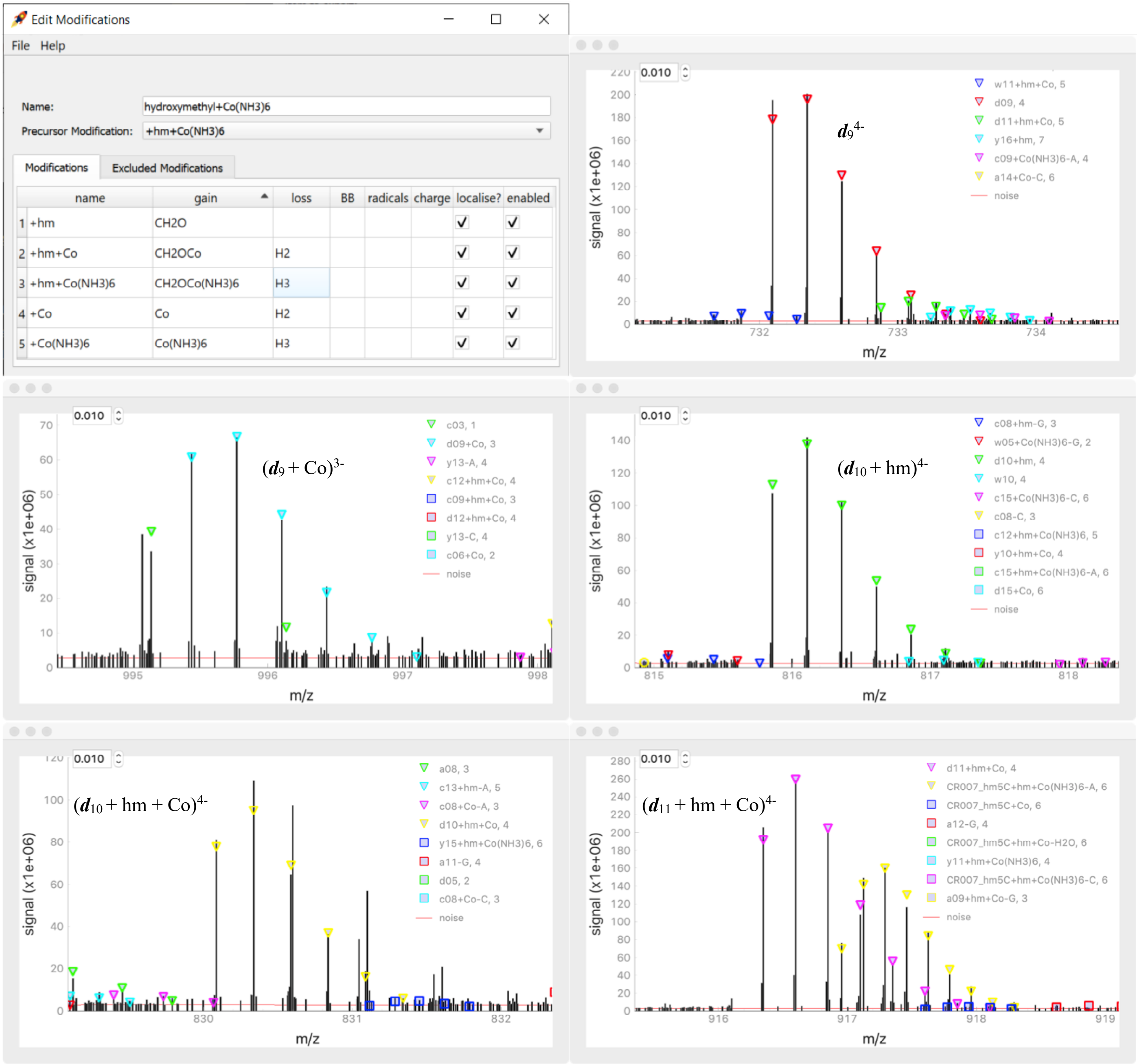

Figure S3: Analysis of multiple modifications by FAST MS. RTD of RNA 3 with a hydroxymethyl (hm) modification (the position of which was 10 but was treated as unknown in the analysis) produced fragments with and without hydroxymethyl (hm) modification and with and without Co attachment. These modifications can be specified in the dialog window 'Edit Modifications' for detection by FAST MS, shown here for shown here for $\boldsymbol{d}_9$, $\boldsymbol{d}_{10}$, and $\boldsymbol{d}_{11}$ fragments as indicated.

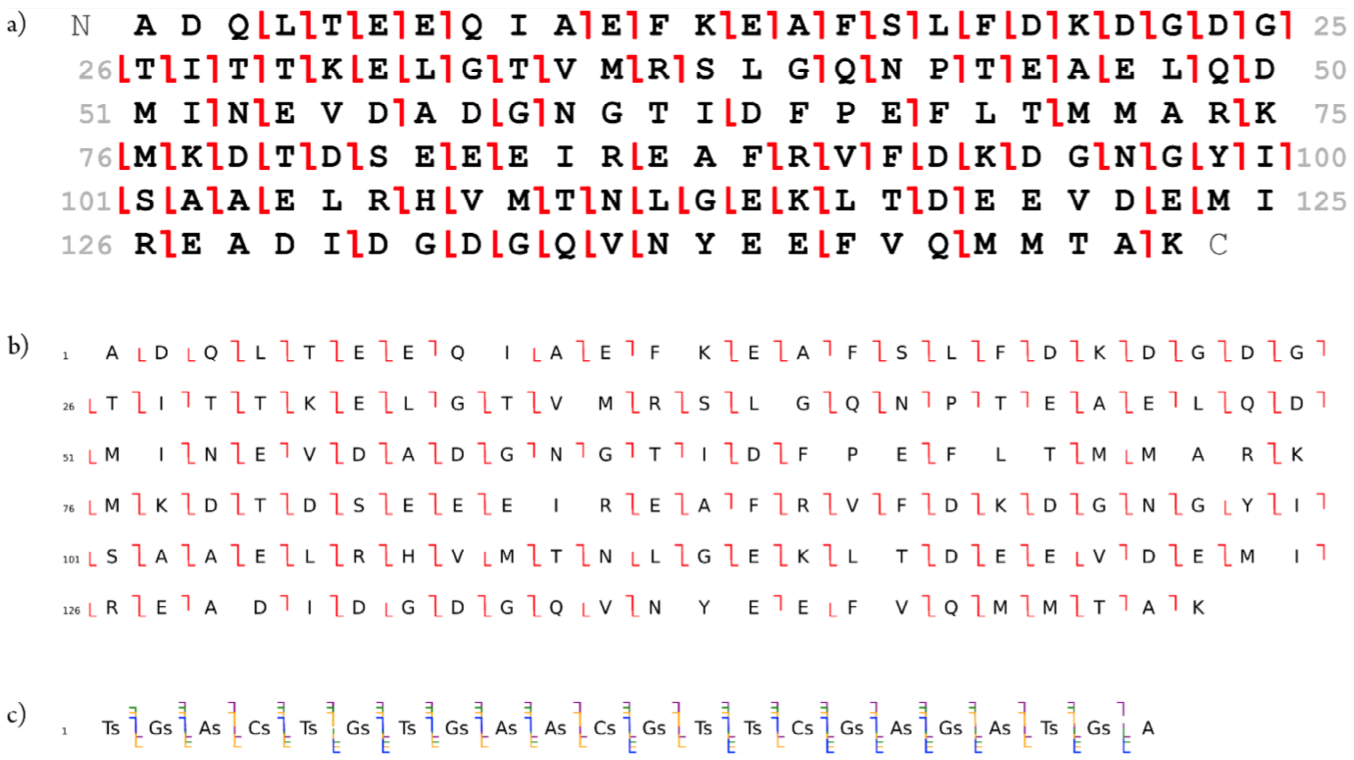

Figure S4: Fragment ion maps illustrating sequence coverage from analysis of an ECD spectrum of calmodulin using a) SNAP and ProSight Lite (64%) and b) FAST MS (87%); c) shows the sequence coverage from analysis of a CAD spectrum of CpG1018 DNA using FAST MS resolved by fragment type (***a***: purple, ***b***: green, ***c***: orange, ***d***: blue, ***w***: purple, ***x***: green, ***y***: orange, ***z***: blue.

Table S1: List of ions assigned by FAST MS followed by manual inspection of the spectrum from CAD of $(NSR^{CMC+}\text{-}8H)^{7-}$ ions shown in Figure 4. Using a S/N threshold of 2, FAST MS identified 482 ions, of which 45 were removed from the list of observed ions and 11 were recovered from the list of deleted ions after manual inspection, resulting in 437 correct assignments. Fragment ions which showed overlap with the far more abundant precursor ions ($c_{11}^{CMC+,3-}$, y_{20}^{5-}, and y_{24}^{6-}) were ignored in the calculation of data for Figures 7b and 8.

m/z (monoisotopic)	z	signal /10^6	name	mass accuracy /ppm	S/N
344.04008	1	10	c_1	-0.23	1.2
538.03832	1	75	c_2-G	0.25	9.2
548.10400	1	1130	y_2	0.82	138.6
610.59798	2	407	y_4	-0.22	41.6
628.06912	1	187	w_2	-1.21	17.9
649.57349	2	785	c_4	0.06	96.3
650.58160	2	283	w_4	0.50	33.9
689.08822	1	561	c_2	0.91	68.8
706.58870	2	272	a_5-G	-0.85	33.4
707.59795	2	25	y_5-C	1.16	3.1
712.09740	3	26	y_7	-2.16	3.1
738.75466	3	252	w_7	0.38	30.9
746.57270	2	155	c_5-G	0.31	19.0
751.41780	3	78	c_7	-0.11	9.6
763.11890	2	2468	y_5	0.19	302.7
766.57434	2	31	c_5-C	-1.57	3.8
782.09590	3	27	y_8-A	-0.88	3.3
790.10097	3	30	y_8-C	0.81	3.7
802.84262	4	81	w_{10}	-1.73	10.0
803.10239	2	401	w_5	0.58	49.1

803.12053	1	94	a_3-C	1.54	11.6
822.09768	2	1910	c_5	0.62	234.3
827.11468	3	806	y_8	-0.09	98.9
831.09467	4	24	a_{11}-C	0.78	3.0
831.35311	4	31	y_{11}-A	1.41	3.9
840.11545	2	42	y_6-G	0.90	5.2
843.07950	1	46	c_3-G	0.03	5.6
847.34631	4	35	w_{11}-G	1.79	4.2
848.11228	2	41	y_6-A	0.15	5.0
851.34316	4	34	w_{11}-A	-0.42	4.2
853.42591	3	328	c_8	-0.48	40.2
853.77036	3	331	w_8	0.19	40.6
859.11140	2	40	a_6-G	1.69	4.9
860.11540	2	87	y_6-C	-2.76	10.7
865.11535	4	265	y_{11}	-0.24	32.5
879.11337	2	173	a_6-C	0.40	21.2
883.08586	1	37	c_3-C	0.27	4.5
885.10772	4	394	w_{11}	0.65	48.3
891.43496	3	138	a_9-G	-2.16	16.9
891.78132	3	245	y_9-G	0.63	30.1
893.15117	1	2874	y_3	0.21	352.5
897.11271	3	126	y_9-A	0.35	15.4
899.09317	2	199	c_6-G	0.07	24.4
905.11547	3	208	y_9-C	-0.75	25.6
909.61590	4	167	y_{12}-G	-0.48	20.5
913.61496	4	228	y_{12}-A	-0.12	28.0
914.16282	1	90	a_3	0.30	11.0
914.30595	5	30	y_{15}-A	0.18	3.7
915.63992	2	4401	y_6	0.56	533.7
918.09201	3	125	c_9-G	-0.35	15.4
919.09625	2	162	c_6-C	0.08	19.9
919.61723	4	106	y_{12}-C	-0.70	13.0
934.63486	2	137	a_6	0.23	16.8
937.15955	3	44	c_8^{CMC+}	-0.02	5.4
941.31677	5	226	y_{15}	0.10	27.7
942.13097	3	5020	y_9	0.43	615.7
947.37900	4	2946	y_{12}	0.32	361.3
955.10710	4	47	c_{12}	0.37	5.2
955.62340	2	115	w_6	0.87	13.6
957.30957	5	172	w_{15}	-0.39	20.7
967.37139	4	164	w_{12}	1.16	13.4
968.44217	3	765	c_9	0.04	60.7
968.78846	3	79	w_9	2.53	7.0
972.11434	5	28	y_{16}-G	-0.74	3.5
973.11577	1	613	w_3	-1.58	75.2
974.61852	2	3724	c_6	0.73	456.7
986.12251	4	276	y_{13}-G	-0.15	33.9
990.12109	4	194	y_{13}-A	-0.31	23.8
993.78899	3	248	y_{10}-G	-0.20	30.4
994.12994	1	1804	c_3	1.07	221.3
996.12359	4	85	y_{13}-C	-0.61	10.4
999.12138	3	112	y_{10}-A	0.55	13.8
1001.12521	2	50	y_7-A	0.41	6.1
1002.32500	5	320	y_{16}	0.06	39.2
1007.12307	3	140	y_{10}-C	-1.49	17.2
1007.55059	5	127	w_{15}^{CMC+}	0.69	13.5
1010.17790	4	87	y_{12}^{CMC+}	-0.73	10.7
1013.13102	2	33	y_7-C	0.60	4.0
1018.31788	5	55	w_{16}	-0.31	6.4

1020.10081	3	234	c_{10}-G	0.05	26.0
1020.44425	3	51	w_{10}-G	-0.38	5.7
1023.88547	4	2710	y_{13}	0.44	332.4
1025.86436	3	103	y_{9}^{CMC+}	0.53	12.7
1030.17056	4	29	w_{12}^{CMC+}	0.34	3.6
1031.61318	4	264	c_{13}	0.10	27.9
1033.12454	5	19	y_{17}-G	1.19	2.1
1036.32038	5	55	y_{17}-A	-1.85	6.7
1043.87713	4	50	w_{13}	0.51	6.0
1044.13912	3	3239	y_{10}	0.12	357.8
1052.10581	2	231	c_{7}-G	0.05	28.4
1052.17631	3	129	c_{9}^{CMC+}	0.88	15.8
1052.56448	5	54	y_{16}^{CMC+}	-0.39	6.5
1062.62850	4	129	y_{14}-G	-0.46	15.9
1063.33223	5	504	y_{17}	-0.90	56.6
1066.62722	4	151	y_{14}-A	-0.47	14.9
1068.65211	2	2491	y_{7}	0.06	268.7
1070.44980	3	1341	c_{10}	-0.71	105.7
1070.79647	3	347	w_{10}	1.89	32.9
1072.10663	2	50	c_{7}-C	-2.05	5.4
1072.63062	4	61	y_{14}-C	0.09	6.6
1079.12002	5	83	c_{17}	-0.10	8.5
1086.68472	4	407	y_{13}^{CMC+}	-0.22	46.0
1087.14886	1	29	y_{4}-A	-0.13	3.1
1087.65123	2	29	a_{7}	3.62	3.1
1090.85660	3	41	y_{10}^{CMC+}-C	-1.13	4.6
1094.32929	5	75	y_{18}-G	0.84	7.7
1094.41207	4	32	c_{13}^{CMC+}	-0.86	3.3
1097.52663	5	94	y_{18}-A	-0.66	8.1
1100.21605	2	136	c_{6}^{CMC+}	-1.49	11.4
1100.39165	4	1441	y_{14}	0.28	143.7
1102.33060	5	22	y_{18}-C	0.91	2.0
1103.47289	3	263	y_{11}-G	-0.44	22.0
1103.83667	3	28	c_{10}^{CMC+}-G	2.41	2.3
1106.67648	4	59	w_{13}^{CMC+}	-0.05	5.3
1108.11964	4	824	c_{14}	0.22	83.0
1108.63622	2	606	w_{7}	0.91	55.3
1108.80453	3	228	y_{11}-A	-0.43	21.5
1110.11410	5	33	c_{18}-G	-1.09	3.2
1113.57413	5	202	y_{17}^{CMC+}	0.88	19.2
1116.80913	3	92	y_{11}-C	0.34	10.1
1121.78246	3	202	c_{11}-G	1.13	14.0
1124.53889	5	579	y_{18}	0.57	58.2
1125.42879	4	59	y_{14}^{CMC+}-G	-0.13	5.7
1127.63112	2	1879	c_{7}	0.58	159.1
1127.87089	3	502	y_{10}^{CMC+}	-1.21	43.4
1129.35931	5	53	c_{17}^{CMC+}	-0.69	4.8
1129.42819	4	82	y_{14}^{CMC+}-A	0.47	7.4
1130.12689	3	58	w_{11}-G	-1.70	5.3
1135.11265	3	50	c_{11}-C	-3.46	4.6
1139.13452	4	167	y_{15}-G	-0.69	13.3
1140.32416	5	207	c_{18}	-0.90	15.6
1143.13374	4	112	y_{15}-A	-0.26	11.3
1144.56695	5	34	y_{18}^{CMC+}-G	-1.20	2.9
1145.47524	3	110	a_{11}	-0.27	9.0
1146.86502	4	144	c_{15}-G	1.43	11.4
1147.76830	5	76	y_{18}^{CMC+}-A	0.87	6.4
1149.10466	1	92	c_{4}-G	-0.10	7.8
1153.82307	3	2491	y_{11}	-0.09	132.8

1154.18480	3	388	$\boldsymbol{c}_{10}^{CMC+}$	0.85	20.3
1159.12834	4	35	$\boldsymbol{w}_{15}$-G	1.25	2.7
1160.35569	5	115	$\boldsymbol{c}_{18}^{CMC+}$-G	0.37	9.0
1163.13731	5	146	$\boldsymbol{a}_{19}$-G	4.97	8.8
1163.19183	4	728	$\boldsymbol{y}_{14}^{CMC+}$	0.47	53.6
1163.33736	5	19	$\boldsymbol{y}_{19}$-G	-0.43	1.4
1165.13033	2	36	$\boldsymbol{a}_{8}$-G	-4.22	3.1
1165.65218	2	121	$\boldsymbol{y}_{8}$-G	0.96	10.3
1166.53664	5	163	$\boldsymbol{y}_{19}$-A	-0.17	12.4
1168.35575	5	90	$\boldsymbol{c}_{18}^{CMC+}$-C	-0.64	6.7
1170.91940	4	185	$\boldsymbol{c}_{14}^{CMC+}$	0.05	16.1
1172.13092	3	1285	$\boldsymbol{c}_{11}$	-0.06	99.7
1173.64536	2	115	$\boldsymbol{y}_{8}$-A	-2.69	6.5
1174.77839	5	559	$\boldsymbol{y}_{18}^{CMC+}$	0.16	35.5
1176.89817	4	809	$\boldsymbol{y}_{15}$	0.43	67.4
1180.47869	3	322	$\boldsymbol{w}_{11}$	0.06	26.7
1184.62545	4	684	$\boldsymbol{c}_{15}$	-0.23	61.1
1185.65560	2	90	$\boldsymbol{y}_{8}$-C	1.24	8.3
1190.56549	5	1084	$\boldsymbol{c}_{18}^{CMC+}$	0.29	71.2
1192.54008	3	32	$\boldsymbol{y}_{11}^{CMC+}$-A	1.53	2.3
1193.54814	5	1406	$\boldsymbol{y}_{19}$	0.33	81.5
1194.25000	2	62	$\boldsymbol{y}_{7}^{CMC+}$	-1.62	3.5
1194.27932	7	151	**NSR**-G-H_2O	-1.16	8.5
1194.86850	7	112	$\boldsymbol{w}_{26}^{CMC+}$-G	-1.76	6.0
1196.56557	7	104	**NSR**-A-H_2O	-0.11	5.3
1196.85099	7	140	**NSR**-G	-2.22	7.0
1196.88904	4	207	$\boldsymbol{w}_{15}$	-0.17	10.4
1197.15720	7	94	$\boldsymbol{w}_{26}^{CMC+}$-A	1.35	4.7
1198.01147	7	234	$\boldsymbol{c}_{26}^{CMC+}$-G	-1.13	13.0
1200.29922	7	110	$\boldsymbol{c}_{26}^{CMC+}$-A	1.17	7.5
1202.49836	6	226	$\boldsymbol{w}_{22}^{CMC+}$	0.10	13.0
1205.02558	7	331	$\boldsymbol{y}_{26}^{CMC+}$	0.22	21.6
1205.11812	2	250	$\boldsymbol{c}_{8}$-G	-0.25	16.7
1205.63483	2	136	$\boldsymbol{w}_{8}$-G	0.50	8.9
1205.93346	4	166	$\boldsymbol{y}_{15}^{CMC+}$-A	-0.44	11.1
1208.82883	6	111	$\boldsymbol{c}_{22}^{CMC+}$	-0.96	5.8
1209.33362	5	174	$\boldsymbol{c}_{19}$	-0.88	8.8
1209.66276	4	150	$\boldsymbol{c}_{15}^{CMC+}$-G	-0.46	7.7
1210.13897	3	62	$\boldsymbol{a}_{12}$-C	-2.14	3.2
1211.15949	7	1054	**NSR**$^{CMC+}$-2G	-1.11	74.1
1213.15861	3	135	$\boldsymbol{y}_{12}$-G	0.88	10.6
1213.44550	7	2510	**NSR**$^{CMC+}$-G-A	-0.27	192.0
1215.73225	7	762	**NSR**$^{CMC+}$-2A	1.18	37.1
1215.85660	7	716	**NSR**-H_2O	-2.14	34.1
1216.44726	7	523	$\boldsymbol{w}_{26}^{CMC+}$	-1.50	24.0
1216.77242	5	166	$\boldsymbol{y}_{19}^{CMC+}$-A	-3.59	8.7
1216.87610	7	2096	**NSR**$^{CMC+}$-G-C	0.08	110.7
1218.43042	7	1250	**NSR**	-1.41	50.1
1218.48951	3	184	$\boldsymbol{y}_{12}$-A	0.27	7.4
1219.16091	7	1644	**NSR**$^{CMC+}$-A-C	-0.06	61.2
1219.59085	7	1363	$\boldsymbol{c}_{26}^{CMC+}$	-0.38	49.3
1222.20327	1	1493	$\boldsymbol{y}_{4}$	-0.19	60.4
1223.46181	3	190	$\boldsymbol{c}_{12}$-G	0.16	9.9
1224.16820	6	104	$\boldsymbol{y}_{23}^{CMC+}$-A	-0.93	5.5
1224.54355	5	337	$\boldsymbol{y}_{20}$-G	0.51	18.0
1225.11920	2	64	$\boldsymbol{c}_{8}$-C	-1.86	3.6
1227.74247	5	336	$\boldsymbol{y}_{20}$-A	0.47	18.3
1227.83442	6	144	$\boldsymbol{a}_{23}^{CMC+}$-C	-0.69	7.8
1229.12463	4	171	$\boldsymbol{c}_{16}$-G	-1.52	8.4

1229.36304	5	346	c_{19}^{CMC+}-G	-1.39	19.7
1230.16549	7	2577	**NSR**$^{CMC+}$-G-H_2O	-0.73	119.0
1230.64112	6	2658	y_{24}-G	0.01	114.5
1232.73773	7	35581	**NSR**$^{CMC+}$-G	-1.30	1593.5
1233.30712	6	1727	y_{24}-A	0.16	60.3
1235.02349	7	27098	**NSR**$^{CMC+}$-A	-0.67	943.4
1237.30985	6	994	y_{24}-C	0.85	11
1237.55284	3	909	y_{11}^{CMC+}	-2.89	10.1
1238.45504	7	14500	**NSR**$^{CMC+}$-C	0.44	307.8
1239.69879	4	853	y_{15}^{CMC+}	0.96	17.4
1241.16119	6	169	c_{23}^{CMC+}-C	-1.45	7.0
1241.17525	2	2778	y_8	-0.41	97.1
1243.78801	5	2302	y_{19}^{CMC+}	0.25	128.8
1246.67727	6	1320	y_{23}^{CMC+}	-0.92	60.6
1247.15412	3	316	a_{12}	-1.49	14.2
1247.42533	4	1473	c_{15}^{CMC+}	-0.27	67.8
1251.74459	7	18050	**NSR**$^{CMC+}$-H_2O	-0.23	1297.8
1253.15628	4	526	y_{16}	-1.36	14.8
1253.23297	2	785	c_7^{CMC+}	2.10	22.1
1254.31548	7	704330	**NSR**$^{CMC+}$	-1.86	19816.7
1255.81571	6	22706	y_{24}	-0.23	131.2
1259.57505	5	2227	c_{19}^{CMC+}	0.33	122.7
1259.67068	6	2458	c_{23}^{CMC+}	0.38	135.4
1263.50752	3	2026	y_{12}	0.14	81.5
1266.88904	4	690	c_{16}	0.15	44.4
1272.50831	6	324	y_{24}^{CMC+}-G	0.45	18.2
1273.81139	3	2010	c_{12}	-0.03	78.1
1274.78301	5	168	y_{20}^{CMC+}-G	0.11	7.6
1275.17543	6	414	y_{24}^{CMC+}-A	1.47	17.1
1275.93475	4	245	a_{16}^{CMC+}-A	0.91	10.3
1277.97991	5	192	y_{20}^{CMC+}-A	-1.52	11.2
1278.19574	4	50	y_{16}^{CMC+}-G	0.13	2.9
1278.67554	6	788	a_{24}^{CMC+}-A	0.04	47.0
1279.17517	6	193	y_{24}^{CMC+}-C	-0.20	11.5
1280.64272	2	1827	c_8	-0.31	149.7
1281.16242	2	291	w_8	2.72	25.9
1282.19371	4	69	y_{16}^{CMC+}-A	-0.47	4.4
1282.38202	5	189	a_{20}^{CMC+}-G	0.82	12.0
1282.78239	5	125	y_{20}^{CMC+}-C	-1.33	7.8
1285.74646	5	139	y_{21}-G	-1.18	10.3
1288.19851	4	48	y_{16}^{CMC+}-C	1.09	3.2
1289.33742	6	790	c_{24}^{CMC+}-G	0.03	52.7
1290.16002	3	59	w_{12}	-2.14	4.0
1291.92263	4	218	c_{16}^{CMC+}-G	-2.95	14.6
1292.00319	6	1072	c_{24}^{CMC+}-A	-0.01	65.6
1293.87366	3	52	a_{12}^{CMC+}-C	-0.89	2.7
1295.92548	4	183	c_{16}^{CMC+}-A	0.24	8.4
1296.00630	6	208	c_{24}^{CMC+}-C	0.95	9.5
1297.68359	6	3124	y_{24}^{CMC+}	0.74	167.4
1298.37417	5	411	c_{20}^{CMC+}-G	-0.05	22.2
1300.15344	1	883	c_4	-0.57	57.4
1301.18674	6	73	a_{24}^{CMC+}	1.67	4.0
1301.56841	5	139	c_{20}^{CMC+}-A	-3.69	7.6
1301.92496	4	0	c_{16}^{CMC+}-C	-2.32	0.0
1302.16912	1	111	w_4	-0.55	6.0
1302.22102	3	69	y_{12}^{CMC+}-A	-1.09	3.8
1304.99290	5	2213	y_{20}^{CMC+}	0.11	188.2
1306.37628	5	52	c_{20}^{CMC+}-C	0.62	3.0
1307.19657	3	121	c_{12}^{CMC+}-G	1.30	7.0

1311.01010	6	258	w_{24}^{CMC+}	-0.20	14.0
1314.51262	6	3858	c_{24}^{CMC+}	0.25	252.3
1315.95724	5	888	y_{16}^{CMC+}	-0.47	19.0
1315.95750	4	309	y_{21}	-0.33	54.5
1320.49724	3	93	y_{13}-A	-0.28	5.6
1320.53113	3	59	c_{12}^{CMC+}-C	0.66	3.4
1323.34926	6	247	y_{25}^{CMC+}-G	0.99	14.8
1325.47028	3	170	c_{13}-G	0.17	8.2
1326.01607	6	222	y_{25}^{CMC+}-A	1.73	11.3
1328.58529	5	1280	c_{20}^{CMC+}	0.88	73.9
1329.41526	4	439	y_{17}	-2.30	26.6
1329.68952	4	2278	c_{16}^{CMC+}	0.55	150.5
1330.89003	3	207	a_{12}^{CMC+}	0.60	11.9
1333.51965	6	220	a_{25}^{CMC+}-G	1.56	13.1
1335.94895	4	178	w_{16}^{CMC+}	-0.42	13.1
1335.98796	5	434	y_{21}^{CMC+}-G	0.02	32.2
1337.65830	2	333	a_{9}-G	-0.49	25.3
1338.17405	2	263	y_{9}-G	-0.54	19.5
1339.18766	5	354	y_{21}^{CMC+}-A	0.56	28.1
1343.98849	5	69	y_{21}^{CMC+}-C	-0.50	5.6
1346.84525	6	1824	c_{25}^{CMC+}-G	-0.03	119.9
1347.24176	3	755	y_{12}^{CMC+}	0.86	50.0
1348.52544	6	1914	y_{25}^{CMC+}	1.92	86.9
1349.14763	4	397	c_{17}	-3.23	21.0
1349.51056	6	979	c_{25}^{CMC+}-A	-0.41	51.5
1351.98007	5	55	w_{21}^{CMC+}-G	-0.84	3.2
1353.51324	6	948	c_{25}^{CMC+}-C	0.19	67.8
1354.45563	4	135	y_{17}^{CMC+}-G	-0.20	9.6
1357.54451	3	1405	c_{12}^{CMC+}	-0.12	86.4
1358.17834	2	123	y_{9}-C	0.36	6.7
1358.19518	4	246	a_{17}^{CMC+}-A	-1.13	13.4
1359.57970	5	842	c_{21}^{CMC+}-G	0.30	58.5
1361.85134	6	239	w_{25}^{CMC+}	0.56	13.6
1362.77807	5	286	c_{21}^{CMC+}-A	-0.15	17.0
1365.51450	3	813	y_{13}	-0.94	57.1
1366.19903	5	2388	y_{21}^{CMC+}	0.89	154.4
1366.77342	2	214	y_{8}^{CMC+}	-1.63	16.0
1367.57851	5	70	c_{21}^{CMC+}-C	-1.47	5.3
1372.02059	6	12254	c_{25}^{CMC+}	0.29	886.9
1374.18979	4	449	c_{17}^{CMC+}-G	0.16	35.1
1374.44051	4	324	w_{17}^{CMC+}-G	-5.07	25.4
1375.82027	3	1012	c_{13}	0.30	68.3
1376.96772	5	118	y_{22}	1.16	8.4
1377.64177	2	122	c_{9}-G	-0.26	8.0
1378.18868	4	340	c_{17}^{CMC+}-A	0.27	23.8
1380.85623	6	641	y_{26}^{CMC+}-G	0.27	44.2
1382.19243	5	182	w_{21}^{CMC+}	0.98	12.2
1383.52295	6	429	y_{26}^{CMC+}-A	0.92	32.5
1384.19299	4	81	c_{17}^{CMC+}-C	1.36	6.2
1384.52548	6	70	a_{26}^{CMC+}-G	2.67	5.8
1387.52332	6	239	y_{26}^{CMC+}-C	-0.16	18.9
1387.89332	4	64	c_{18}-G	-1.90	4.9
1389.78955	5	3257	c_{21}^{CMC+}	0.26	255.8
1392.21848	4	1166	y_{17}^{CMC+}	0.17	96.4
1396.99688	5	179	y_{22}^{CMC+}-G	0.49	11.0
1398.90015	3	40	y_{13}^{CMC+}-G	0.66	3.3
1400.19581	5	168	y_{22}^{CMC+}-A	0.45	16.1
1404.59626	5	77	a_{22}^{CMC+}-G	1.40	6.1
1404.99762	5	51	y_{22}^{CMC+}-C	0.14	4.0

1406.03040	6	3153	$y_{26}$$^{CMC+}$	-0.25	186.1
1406.24397	2	1245	$c_{8}$$^{CMC+}$	0.69	73.5
1409.20211	3	201	$c_{13}$$^{CMC+}$-G	-0.85	13.6
1409.69919	6	74	$a_{26}$$^{CMC+}$	1.77	5.2
1411.95119	4	2906	$c_{17}$$^{CMC+}$	-0.52	227.5
1413.70055	2	1944	y_{9}	0.75	116.6
1414.18035	1	174	a_{5}-G	-3.91	10.5
1416.20613	1	70	y_{5}-C	3.25	4.7
1417.17850	3	65	y_{14}-G	2.89	4.4
1420.58784	5	385	$c_{22}$$^{CMC+}$-G	0.20	29.5
1422.50159	3	34	y_{14}-A	-3.13	2.6
1423.78418	5	152	$c_{22}$$^{CMC+}$-A	-1.66	11.0
1425.65399	4	136	c_{18}	-3.03	12.2
1427.20615	5	1205	$y_{22}$$^{CMC+}$	0.05	85.9
1428.58949	5	104	$c_{22}$$^{CMC+}$-C	0.49	8.0
1430.96267	4	38	$y_{18}$$^{CMC+}$-G	0.31	3.9
1434.80815	5	134	$a_{22}$$^{CMC+}$	2.77	13.6
1434.95977	4	41	$y_{18}$$^{CMC+}$-A	-0.82	4.2
1443.19822	5	268	$w_{22}$$^{CMC+}$	-0.77	23.1
1445.97406	5	296	y_{23}	-1.07	22.1
1449.24835	3	468	$y_{13}$$^{CMC+}$	-0.47	38.4
1450.69679	4	692	$c_{18}$$^{CMC+}$-G	0.62	46.3
1450.79801	5	1714	$c_{22}$$^{CMC+}$	0.39	117.1
1453.16617	2	468	c_{9}	-0.46	30.5
1454.19781	1	61	a_{5}-C	3.98	4.2
1454.41661	4	106	y_{19}-G	-5.18	7.0
1454.69674	4	395	$c_{18}$$^{CMC+}$-A	1.46	30.2
1458.42353	4	44	y_{19}-A	0.46	4.5
1459.55295	3	1428	$c_{13}$$^{CMC+}$	-0.11	134.0
1460.69592	4	171	$c_{18}$$^{CMC+}$-C	-1.04	15.5
1463.25881	2	144	$a_{9}$$^{CMC+}$-G	-0.03	14.8
1466.00555	5	199	$y_{23}$$^{CMC+}$-G	-0.09	15.0
1467.52344	3	300	y_{14}	-0.52	17.3
1468.72458	4	379	$y_{18}$$^{CMC+}$	0.01	21.8
1469.20571	5	114	$y_{23}$$^{CMC+}$-A	0.71	7.0
1473.60359	5	143	$a_{23}$$^{CMC+}$-C	-0.13	14.3
1474.00448	5	72	$y_{23}$$^{CMC+}$-C	-1.65	7.2
1477.82855	3	473	c_{14}	0.18	39.5
1481.59564	5	287	$c_{23}$$^{CMC+}$-G	-0.11	23.8
1484.78977	5	60	$c_{23}$$^{CMC+}$-A	-3.38	5.7
1488.45774	4	4458	$c_{18}$$^{CMC+}$	-0.34	435.3
1491.18123	2	81	y_{10}-G	-4.16	7.6
1492.18384	4	404	y_{19}	-1.78	35.6
1496.21361	5	1097	$y_{23}$$^{CMC+}$	-1.30	98.5
1500.90986	3	49	$y_{14}$$^{CMC+}$-G	1.46	4.6
1503.24334	2	117	$c_{9}$$^{CMC+}$-G	0.89	12.0
1511.20845	3	454	$c_{14}$$^{CMC+}$-G	-2.18	35.6
1511.80463	5	677	$c_{23}$$^{CMC+}$	-0.70	54.4
1512.20563	5	177	$w_{23}$$^{CMC+}$	-2.11	14.3
1516.96486	4	289	$a_{19}$$^{CMC+}$-G	-0.87	30.4
1519.18239	3	51	y_{15}-G	-0.30	6.0
1521.22255	4	82	$y_{19}$$^{CMC+}$-A	-0.17	9.3
1523.24717	2	51	$c_{9}$$^{CMC+}$-C	1.37	5.4
1524.54497	3	92	$c_{14}$$^{CMC+}$-C	-1.41	9.0
1527.24415	1	354	y_{5}	-0.42	43.4
1529.48756	3	56	c_{15}-G	0.42	6.1
1530.65458	2	70	c_{10}-G	-0.13	7.1
1534.61125	5	89	$a_{24}$$^{CMC+}$-A	-0.51	8.7
1534.90713	3	67	$a_{14}$$^{CMC+}$	0.67	6.5

1536.95700	4	451	c_{19}^{CMC+}-G	-0.49	51.3
1539.29822	2	282	y_{9}^{CMC+}	-0.75	28.3
1540.95748	4	63	c_{19}^{CMC+}-A	0.65	5.9
1541.20842	4	47	w_{19}^{CMC+}-A	-3.88	4.4
1547.40748	5	54	c_{24}^{CMC+}-G	0.75	6.0
1550.61005	5	34	c_{24}^{CMC+}-A	3.07	3.2
1551.25923	3	338	y_{14}^{CMC+}	1.14	31.9
1551.35272	1	45	c_{4}^{CMC+}	-0.79	4.3
1554.98689	4	505	y_{19}^{CMC+}	0.29	44.6
1557.42083	5	110	y_{24}^{CMC+}	0.14	8.7
1561.56136	3	4372	c_{14}^{CMC+}	-0.12	422.1
1565.23478	1	82	a_{5}	-0.32	6.0
1566.71071	2	404	y_{10}	-0.91	26.6
1568.69048	4	55	y_{20}	-1.49	4.4
1569.53195	3	229	y_{15}	-0.44	18.6
1574.72082	4	1045	c_{19}^{CMC+}	0.45	93.0
1577.61644	5	190	c_{24}^{CMC+}	0.16	17.1
1578.76481	2	505	c_{9}^{CMC+}	-1.20	42.6
1579.83794	3	99	c_{15}	0.77	9.5
1593.72902	4	44	y_{20}^{CMC+}-G	-0.87	5.4
1596.19217	3	45	w_{15}	2.56	5.5
1603.22707	4	152	a_{20}^{CMC+}-G	-0.60	18.1
1606.17786	2	163	c_{10}	-1.01	17.5
1607.21321	1	45	w_{5}	1.30	4.6
1613.21894	3	388	c_{15}^{CMC+}-G	-0.77	47.6
1618.42859	5	24	y_{25}^{CMC+}	-0.18	2.9
1623.21967	4	73	c_{20}^{CMC+}-G	0.03	8.9
1626.19102	3	19	y_{16}-A	-2.39	2.3
1626.55576	3	85	c_{15}^{CMC+}-C	0.12	10.2
1631.49486	4	130	y_{20}^{CMC+}	1.29	15.3
1636.91476	3	32	a_{15}^{CMC+}	0.14	3.9
1645.19382	1	36	c_{5}	-4.74	3.8
1651.48060	4	66	w_{20}^{CMC+}	-2.27	7.2
1653.26632	3	186	y_{15}^{CMC+}	0.25	17.6
1655.70788	2	41	y_{11}-G	-3.51	4.5
1656.25518	2	57	c_{10}^{CMC+}-G	0.32	6.4
1660.98122	4	129	c_{20}^{CMC+}	-0.45	15.9
1663.56909	3	2185	c_{15}^{CMC+}	-0.54	221.0
1671.20854	3	57	y_{16}	-2.72	6.1
1679.92370	3	20	w_{15}^{CMC+}	1.40	2.5
1692.31327	2	102	y_{10}^{CMC+}	0.74	12.5
1696.24893	3	39	a_{16}^{CMC+}-G	0.02	4.8
1701.58021	3	224	a_{16}^{CMC+}-A	-0.20	27.5
1707.99731	4	58	y_{21}^{CMC+}	-1.04	7.1
1709.57568	3	19	a_{16}^{CMC+}-C	-5.03	2.3
1722.89671	3	89	c_{16}^{CMC+}-G	-4.43	10.3
1728.23586	3	45	c_{16}^{CMC+}-A	-0.07	5.5
1731.23143	2	87	y_{11}	-4.03	10.7
1731.77957	2	325	c_{10}^{CMC+}	0.12	39.9
1737.48236	4	31	c_{21}^{CMC+}	-3.41	3.8
1754.94643	3	52	y_{16}^{CMC+}	0.05	6.3
1772.88724	3	47	y_{17}	-3.53	5.6
1773.25522	3	252	c_{16}^{CMC+}	0.60	28.9
1781.59207	3	25	w_{16}^{CMC+}	-5.45	3.1
1808.77023	2	30	c_{11}^{CMC+}-G	-2.81	3.7
1811.25822	3	25	a_{17}^{CMC+}-A	-3.59	3.1
1832.28365	1	55	y_{6}	-1.33	6.7
1832.58592	3	27	c_{17}^{CMC+}-G	-1.42	3.3
1882.93346	3	27	c_{17}^{CMC+}	-2.59	3.3

1884.30082	2	168	$\boldsymbol{c}_{11}^{CMC+}$	0.43	20.6
1935.75976	2	31	$\boldsymbol{w}_{12}$	6.17	3.8
1953.82883	2	24	$\boldsymbol{y}_{12}^{CMC+}$-A	-4.33	2.9
1996.84125	2	25	$\boldsymbol{a}_{12}^{CMC+}$	1.88	3.1

Table S2: Comparison of the performance of FAST MS and SNAP in the assignment of ions using five spectra from CAD of RNA. Incorrect assignments by FAST MS were identified by manual inspection of the spectrum.

RNA	modification	charge state	# ions assigned by SNAP	# ions assigned by FAST MS	ratio # assignments FAST MS/ SNAP	incorrect, FAST MS	% false positives, FAST MS
NSR	$C_5H_{10}O_4$	6-	97	227	2.3	8	4
NSR	$C_5H_{10}O_4$	6-	67	192	2.9	7	4
NSR	CMC^+	7-	83	191	2.3	18	9
NSR	CMC^+	7-	113	232	2.1	12	5
RRE-IIB-0	CMC^+	10-	153	376	2.5	7	2

Table S3: Summary of experiments for estimating the false discovery rate (FDR) of ion assignments by FAST MS.

Precursor	molecule	net charge	dissociation technique	mass /kDa	S/N thres.	quality error thres.	fragments evaluated	ions found with correct sequence	fake sequence	mass (fake sequence) /kDa	ions found with fake sequence	FDR /%
RNA 1	RNA	-4	CAD	4.8	2.0	0.5	***c, y***	29	**$(A)_{15}$**	4.9	1	3
RNA 1^{CMC+}	RNA	-4	CAD	5.0	2.0	0.5	***c, y***	37	**$(A)_{15}^{CMC+}$**	5.1	2	5
RNA 2	RNA	-4	CAD	4.8	2.0	0.5	***c, y***	31	**$(A)_{15}$**	4.9	2	6
RNA 4^{CMC+}	RNA	-5	CAD	7.3	2.0	0.5	***c, y***	67	**$(A)_{22}^{CMC+}$**	7.4	1	1
RNA 5	RNA	-5	CAD	7.0	2.0	0.5	***c, y***	48	**$(A)_{22}$**	7.2	2	4
NSR^{CMC+}	RNA	-6	CAD	8.8	2.0	0.5	***c, y***	98	**$(A)_{27}^{CMC+}$**	9.1	2	2
NSR^{CMC+}	RNA	-6	CAD	8.8	2.0	0.5	***c, y***	89	**$(A)_{27}^{CMC+}$**	9.1	5	6
NSR^{CMC+}	RNA	-7	CAD	8.8	2.0	0.5	***c, y***	117	**$(A)_{27}^{CMC+}$**	9.1	6	5
RRE-IIB-0^{CMC+}	RNA	-9	CAD	12.9	2.0	0.5	***c, y***	173	**$(A)_{39}^{CMC+}$**	13.0	22	13
RRE-IIB-0^{CMC+}	RNA	-10	CAD	12.9	2.0	0.5	***c, y***	224	**$(A)_{39}^{CMC+}$**	13.0	17	8
RRE-I^{CMC+}	RNA	-10	CAD	15.4	2.0	0.5	***c, y***	163	**$(A)_{47}^{CMC+}$**	15.7	12	7
RRE-I^{CMC+}	RNA	-11	CAD	15.4	2.0	0.5	***c, y***	193	**$(A)_{47}^{CMC+}$**	15.7	23	12
ubiquitin	protein	8	ECD	8.6	3.0	0.5	***c, z***	397	**truncated calmodulin**	8.4	21	5
ubiquitin	protein	8	ECD	8.6	3.0	0.5	***c, z***	282	**truncated calmodulin**	8.4	8	3
ubiquitin	protein	11	ECD	8.6	3.0	0.5	***c, z***	387	**truncated calmodulin**	8.4	6	2
calmodulin	protein	16	ECD	16.7	3.0	0.5	***c, z***	440	**extended ubiquitin**	16.7	53	12
calmodulin	protein	16	CAD	16.7	3.0	0.5	***b, y***	232	**extended ubiquitin**	16.7	41	18
DNA 1	DNA	-9	CAD	6.0	2.0	0.5	***a***, ***a***-B, ***w***	89	**$(A)_{20}$**	6.2	0	0
DNA 1	DNA	-9	EDD	6.0	2.0	0.5	***d, w***	39	**$(A)_{20}$**	6.2	1	3
CpG1018	DNA	-9	CAD	7.1	5.0	0.3	***a***, ***a***-B, ***b, c, d, w, x, y, z***	342	**$U(A)_{20}U$**	7.1	7	2
